# Simulation-Guided Interpretable Fault Diagnosis of Hydraulic Directional Control Valves Under Limited Fault Data Conditions

**DOI:** 10.3390/s26072052

**Published:** 2026-03-25

**Authors:** Yuxuan Xia, Aiping Xiao, Huafei Xiao, Xiangyi Zhao, Huijun Liu

**Affiliations:** School of Technology, Beijing Forestry University, Beijing 100083, China; iris918919@bjfu.edu.cn (Y.X.); xhflq@126.com (H.X.); xiangyi9527@bjfu.edu.cn (X.Z.);

**Keywords:** hydraulic directional control valve, fault diagnosis, simulation-guided feature construction, interpretable diagnostics

## Abstract

Delayed switching faults in hydraulic directional control valves can significantly degrade system performance and reliability, yet their diagnosis remains challenging due to complex fault mechanisms and coupled sensor responses and limited fault samples in industrial applications. While data-driven approaches, including deep learning-based methods, have shown promising performance in fault diagnosis, their practical deployment in industrial quality inspection and condition monitoring is often constrained by limited fault data availability and insufficient physical interpretability of the diagnostic results. In this study, an interpretable fault diagnosis framework for delayed switching faults in hydraulic directional control valves is proposed based on a simulation-guided feature construction method and multi-pressure signal analysis. Instead of using simulation to generate synthetic training data, a physical simulation model is employed to analyze fault mechanisms and to guide the design of valve-level diagnostic features derived from inter-sensor pressure differences. These features are further evaluated using several classical machine learning classifiers, including RF, SVM, KNN, and LR under conditions of limited fault samples. Experimental results demonstrate that the proposed method effectively captures the structural imbalance caused by internal valve faults and achieves high diagnostic accuracy and robustness compared with conventional single-sensor approaches and purely data-driven black-box models. The proposed framework provides a practical and physically interpretable solution for hydraulic valve fault diagnosis under small-sample conditions and offers potential value for industrial quality inspection and maintenance applications.

## 1. Introduction

Delayed switching faults in hydraulic directional control valves (DCVs) represent a critical source of performance degradation and reliability loss in electro-hydraulic systems. Such faults typically manifest as prolonged actuation delays, abnormal pressure transients, and asymmetric flow responses, which directly impair system stability and control accuracy. Hydraulic valves are essential components in modern electro-hydraulic equipment used in industrial automation, transportation, and energy systems, and their operational reliability directly influences the overall system performance [1]. However, accurate fault diagnosis for hydraulic components remains challenging due to strong coupling among mechanical, hydraulic, and electromagnetic subsystems, as well as the limited availability of labeled fault samples in industrial environments [2,3,4].

Existing fault diagnosis approaches for hydraulic systems can generally be categorized into traditional signal-processing-based methods and data-driven learning-based methods. Traditional diagnostic approaches rely on handcrafted features extracted from measured signals using time-domain, frequency-domain, or time–frequency analysis techniques. Typical feature extraction methods include envelope spectrum analysis, spectral correlation, entropy-based descriptors, and other statistical signal analysis approaches. For instance, Chen et al. proposed a product envelope spectrum generated from spectral correlation/coherence to enhance impulsive fault features in bearing fault diagnosis, demonstrating strong robustness under noisy conditions [5]. Similar signal-processing-based methods have also been applied to hydraulic systems. Zhao et al. utilized time–frequency analysis techniques to extract diagnostic features from hydraulic component signals [6], while Zheng et al. proposed entropy-based feature extraction methods to characterize hydraulic fault signatures [7]. These approaches are valued for their clear physical interpretability and low computational complexity. However, their diagnostic performance often depends heavily on expert knowledge and carefully designed feature extraction strategies, which may limit their adaptability under varying operating conditions

With the rapid development of artificial intelligence technologies, data-driven fault diagnosis methods have been increasingly applied to hydraulic systems. Classical machine learning algorithms such as support vector machines and Bayesian inference and adaptive fuzzy logic have been used to identify hydraulic component faults and implement fault-tolerant control strategies [3,8,9,10]. For example, Tong and Sepehri applied a SaRT–SVM algorithm to recognize leakage patterns in hydraulic check valves [11]. In addition, multi-pressure signal analysis methods have been developed to capture dynamic interactions between different valve ports for fault identification [12,13,14]. These approaches provide improved diagnostic performance compared with purely signal-processing-based techniques.

More recently, deep learning methods have significantly advanced fault diagnosis capabilities by automatically learning discriminative representations from raw signals. Various neural network architectures, including convolutional neural networks, autoencoders, and fully convolutional networks, have been applied to hydraulic systems and industrial equipment for fault classification [15,16,17]. Transformer-based architectures have also been introduced to capture long-range temporal dependencies in hydraulic pressure signals [18,19]. In addition, deep learning techniques have been applied to related electromechanical equipment and industrial devices, including converter valve systems and complex machinery [20,21]. While these methods can achieve high diagnostic accuracy under sufficient training data, they often require large datasets and involve substantial computational complexity due to deep neural network architectures [22].

To address the data scarcity problem commonly encountered in industrial applications, several studies have explored hybrid model-data-driven diagnostic strategies. For example, Qiu et al. proposed a physical model and Bayesian theory-based fault diagnosis framework for hydraulic components [8], while Shao et al. developed a generative adversarial network-based approach to improve diagnostic performance under limited fault samples [23]. In addition, recent research has introduced physics-informed deep learning models that integrate physical constraints into neural network training processes [24]. These approaches improve generalization capability by incorporating prior knowledge of system dynamics.

Another emerging direction involves simulation-enhanced and digital twin-based fault diagnosis frameworks. Digital twin models can generate high-fidelity virtual data that supplement limited experimental datasets and facilitate cross-domain learning. For example, Yang et al. proposed a digital twin-assisted fault diagnosis method for hydraulic systems [25]. Similarly, Qiao et al. developed a digital twin-guided physical–virtual denoising method for early fault detection of rolling element bearings [26]. Ning et al. further introduced a digital twin-driven cross-domain adaptation framework to transfer diagnostic knowledge between simulated and real bearing systems under varying operating conditions [27]. These approaches demonstrate that simulation models can effectively enhance fault diagnosis performance by generating synthetic training data for data-driven models.

Despite these advances, most existing digital twin-based or deep learning-based approaches focus primarily on rotating machinery such as bearings and rely on complex neural network architectures. In addition, their diagnostic interpretability often remains limited because fault features are implicitly embedded within deep model representations. From an industrial perspective, there remains a strong demand for lightweight, interpretable, and computationally efficient diagnostic methods that can operate reliably under limited fault samples.

To address these challenges, this study proposes a simulation-guided interpretable fault diagnosis framework for hydraulic directional control valves. Unlike existing digital twin-driven approaches that use simulation models to generate training datasets, the proposed method employs simulation primarily as a mechanism-analysis tool. A physically grounded hydraulic simulation model is used to analyze pressure coupling behaviors among valve ports under delayed switching faults. Based on these insights, interpretable diagnostic features are constructed using time-series distance measures and statistical descriptors of inter-port pressure differences.

As illustrated in Table 1, by combining simulation-guided feature construction with classical machine learning classifiers, the proposed framework achieves reliable fault diagnosis with minimal computational complexity and a small number of training samples. Compared with recent deep learning approaches for hydraulic systems [17,18,19,20,24] and data-intensive multimodal diagnostic frameworks [28], the proposed method provides a lightweight and interpretable alternative that is particularly suitable for industrial quality inspection and condition monitoring applications where transparency, robustness, and deployability are essential.

## 2. Materials and Methods

### 2.1. Model Construction

**Figure 1:**
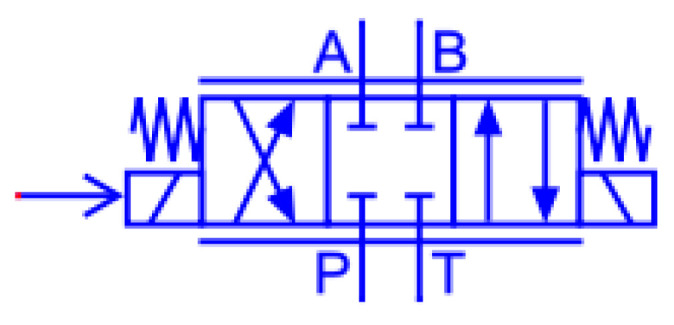
Schematic Diagram of a Three-position Four-way Valve. The arrows indicate flow paths and spool positions, while P, T, A, and B denote standard hydraulic ports.

**Figure 2:**
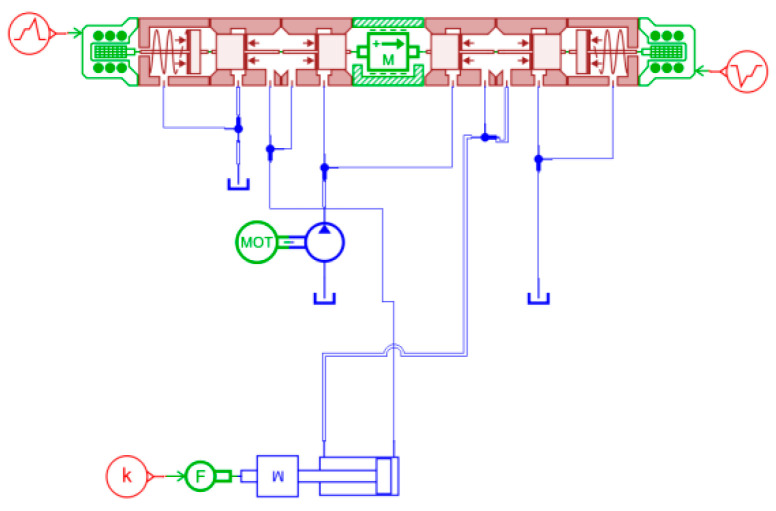
Simulation Oil Circuit Diagram. Blue lines and components represent hydraulic flow paths and the actuator (cylinder); red regions indicate the internal structure of the simulated hydraulic valve and its signal input interface; green elements denote damping units, the pump motor, and the module that converts electrical signals into electromagnetic forces for valve actuation.

The delayed switching behavior of hydraulic directional control valves is primarily attributed to three typical fault mechanisms: degradation of the return spring stiffness due to long-term mechanical fatigue, partial blockage of valve orifices caused by particulate contamination in hydraulic fluid, and weakening of electromagnetic force resulting from magnetic core aging during prolonged operation. To analyze the dynamic characteristics induced by these fault mechanisms, a simulation model was constructed in the AMESim environment and used exclusively for physical mechanism analysis and feature design guidance. A three-position, four-way solenoid directional control valve was selected as the simulation subject, with its principle illustrated in Figure 1. Since the built-in valve components in AMESim do not support parameter-level fault manipulation, customized functional modules were developed based on hydraulic valve operating principles to enable controllable fault modeling. The principle is illustrated in Figure 2, primarily comprising the following modules: 1: Signal Input Module 2: Signal Conversion Module 3: Spring 4: Spool and Valve Body 5: Simulated Damping 6: Reservoir and Motor 7: Hydraulic Cylinder. The simulation workflow consists of signal-to-force conversion, spool actuation driven by spring force, and subsequent hydraulic flow redistribution, which collectively reproduce the directional switching dynamics of the valve. The diagram illustrates the distinct positions of the centre-position ABPT. When the force on the left spring exceeds resistance, the entire spool moves rightward, shifting to the left position: P connects to B, T connects to A. Fluid enters the cylinder through its right port, driving it leftward. Conversely, force applied to the cylinder’s right side overcomes resistance, pushing the spool leftward to connect AP and BT, causing the hydraulic cylinder to reverse direction.

The entire process is simulated in three stages to replicate the hydraulic valve’s movement from centre position to left position, then to right position, and finally back to left position. Overall parameter settings are detailed in Table 2.

#### Simulation Model Validation and Accuracy Assurance

To ensure the reliability and physical credibility of the simulation results, multiple validation measures were implemented for the AMESim-based electro-hydraulic valve model.

First, key physical parameters—including valve geometric dimensions, spool mass, spring stiffness, and electromagnetic actuator characteristics—were determined based on manufacturer technical specifications and design references. Frictional and damping-related parameters, such as Coulomb friction, static friction, and viscous coefficients, were identified through experimental calibration and simulation tuning within physically reasonable ranges. In addition, hydraulic supply pressure, oil properties, and load boundary conditions were configured in accordance with the physical test bench to ensure realistic operating constraints.

Second, simulation outputs were qualitatively compared with experimentally measured pressure signals under healthy operating conditions. Particular attention was given to transient switching characteristics, including response delay, pressure overshoot, and steady-state stabilization behavior. The simulation curves exhibited consistent dynamic trends and temporal evolution patterns with the measured signals, indicating that the model adequately captures the essential pressure coupling mechanisms of the valve system.

Third, it is emphasized that simulation data were not used for classifier training, testing, or data augmentation. Instead, simulations were employed solely for mechanism exploration and physics-informed feature design. Therefore, the diagnostic performance of the proposed framework does not depend on numerical matching between simulated and experimental datasets, but rather on experimentally validated, physically interpretable pressure-response characteristics.

Through parameter configuration grounded in physical specifications, calibration-assisted tuning, and qualitative signal consistency verification, the simulation model provides a physically credible and methodologically reliable foundation for the proposed simulation-guided feature construction framework.

### 2.2. Fault Types and Physical Modeling

This study considers three representative delayed switching fault modes commonly observed in industrial plate-mounted, three-position, four-way solenoid directional control valves, namely spring degradation, spool orifice blockage, and magnetic core loss. Each fault is modeled in the AMESim simulation environment through physically grounded, parameterized adjustments that enable controlled and repeatable emulation of real-world failure mechanisms.

**Figure 3:**
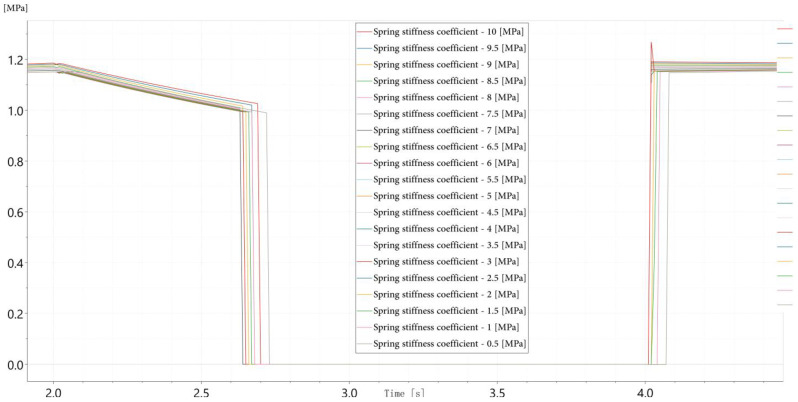
Combined Diagram of Pressure Changes Corresponding to Spring Loss.

**Figure 4:**
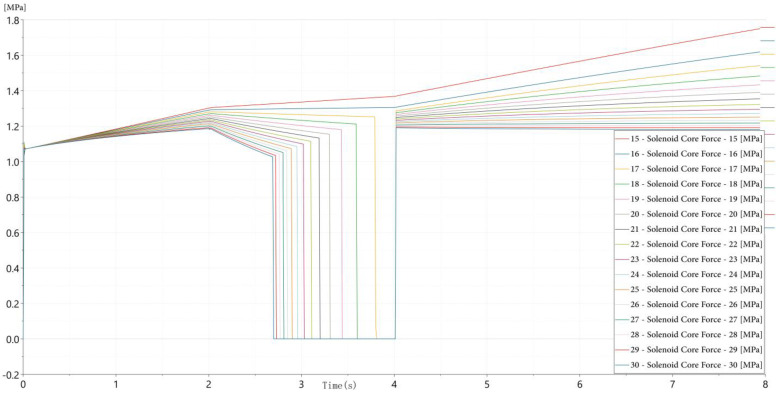
Combined Diagram of Pressure Changes Corresponding to Magnetic Core Loss.

**Figure 5:**
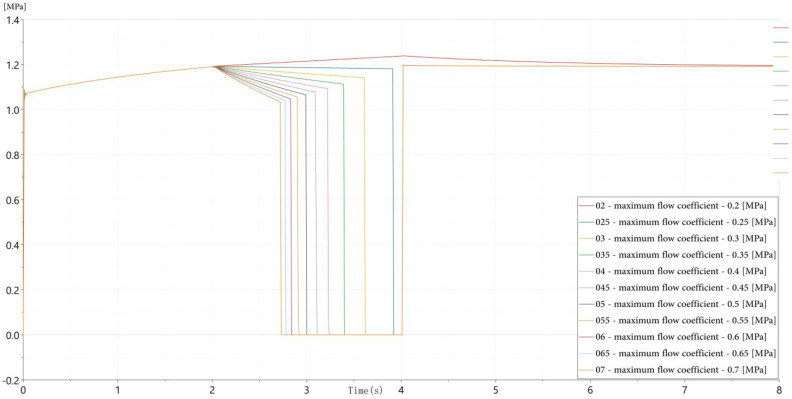
Combined Diagram of Pressure Changes Corresponding to Valve Core Blockage.

(1) Spring Degradation: Prolonged mechanical cycling causes fatigue in the return spring, reducing its elastic stiffness and slowing the spool’s return motion. As shown in Figure 3, the outlet pressure response exhibits progressively increased delay and slower decay during the valve closing phase as stiffness decreases, which is consistent with the expected dynamic behavior associated with mechanical wear.

(2) Spool Orifice Blockage: Contaminant particles in the hydraulic fluid can accumulate at the valve ports, partially obstructing flow paths and increasing flow resistance. This fault is emulated by reducing the effective orifice area of the spool. Figure 4 illustrates that increasing blockage levels lead to slower pressure rise rates and extended time-to-steady-state during valve opening, reflecting the increased hydraulic impedance caused by restricted flow paths.

(3) Magnetic Core Loss: Extended operation may cause demagnetization or thermal aging of the solenoid’s magnetic core, weakening the electromagnetic force and delaying actuation. Figure 5 demonstrates that reduced excitation current is used as an equivalent and practically feasible representation of magnetic core degradation, resulting in longer actuation delays and reduced initial pressure rise slopes. This modeling strategy reflects the attenuation of electromagnetic force and is widely adopted in hydraulic valve diagnostics, where abnormal current characteristics are commonly employed to characterize electromagnetic performance degradation without physically damaging the solenoid structure [32].

These three fault models collectively represent distinct failure domains—mechanical, fluidic, and electromagnetic—and produce distinguishable dynamic signatures in pressure signals. The parameter variation ranges adopted in the simulation primarily aim to capture relative fault-induced trends in pressure responses, providing a mechanism-oriented basis for subsequent feature construction rather than a direct numerical alignment with experimental degradation severity.

It should be explicitly clarified that the numerical simulation in this study is not intended to quantitatively reproduce the exact fault severity levels observed in the experiments, nor to generate training or testing datasets for the diagnostic model. Instead, the simulation model is employed solely as a qualitative analysis tool to investigate fault mechanisms and to identify characteristic pressure response trends associated with different degradation modes of the hydraulic directional control valve. All diagnostic features, model training, and performance evaluation are conducted exclusively using experimentally measured pressure signals. By strictly separating simulation-based mechanism analysis from data-driven fault diagnosis, the proposed framework avoids information leakage and ensures that the diagnostic performance is not biased by simulated data. It should be noted that different fault emulation strategies were adopted for different fault types. Magnetic core degradation was emulated by reducing the drive current, whereas spring degradation and spool blockage were implemented through physical modifications. This discrepancy introduces a degree of inconsistency between simulated and experimental fault representations. However, the reduced-current strategy serves as a conservative proxy for magnetic force loss and does not artificially enhance fault separability. This limitation is acknowledged and will be addressed in future work through more unified fault emulation schemes.

### 2.3. Introduction to the Oil Circuit Dataset

**Figure 6:**
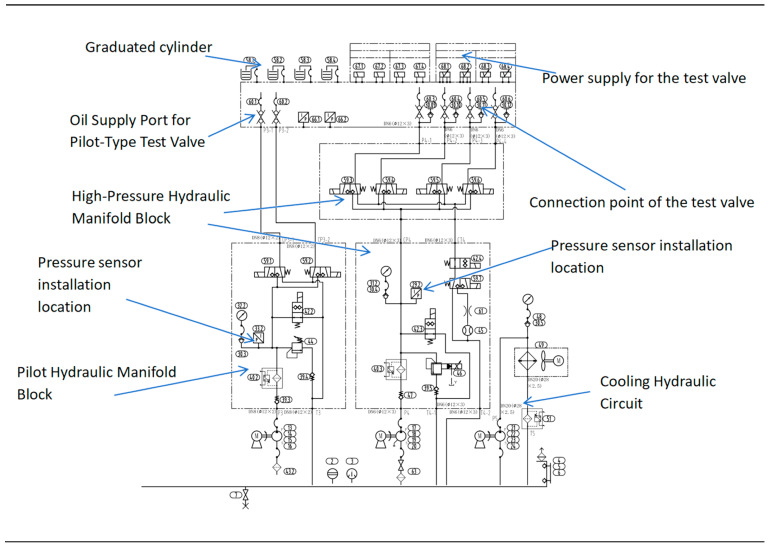
The hydraulic test system schematic includes pilot oil circuits and pressure-resistant oil circuits.

**Figure 7:**
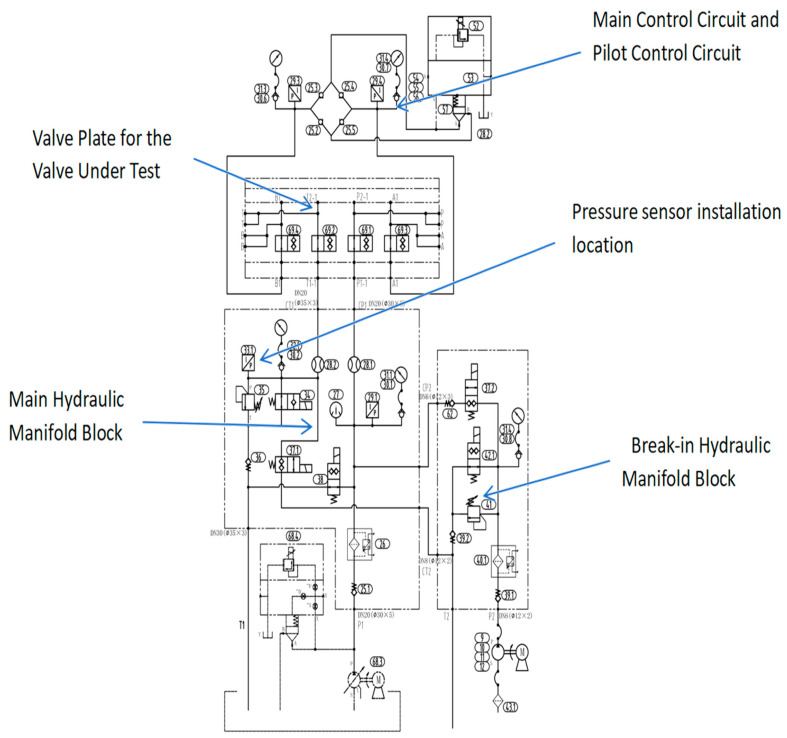
The hydraulic test system schematic includes the break-in test oil circuit and the main oil circuit.

The experimental data used in this study were collected from a dedicated hydraulic valve comprehensive performance test bench (Tianmin Hydraulic Valve Semi-automatic Test Rig, manufactured by Beijing Changzheng Tianmin Co., Ltd., Beijing, China). As illustrated in Figure 6 and Figure 7, the test rig integrates multiple independent hydraulic circuits, including a main test circuit, a break-in circuit, a pilot circuit, and a high-pressure leakage test circuit. For all fault injection and data acquisition experiments reported in this work, the main test circuit was employed to supply pressurized oil to the Device Under Test (DUT). This circuit is capable of delivering a continuously adjustable pressure up to 32 MPa and a flow rate up to 150 L/min, controlled via a variable-frequency drive and a proportional relief valve. Throughout the experiments, the supply pressure was stably maintained at 21 MPa, a representative operating condition aligned with the acceptance testing requirements for aerospace hydraulic components.

Two representative plate-mounted, three-position, four-way solenoid directional control valves were selected as the DUT to validate the generality of the proposed fault diagnosis framework:

Rexroth 4WE10H3X/CG24N9K4 (Bosch Rexroth AG, Lohr am Main, Germany): This valve features an H-type spool configuration (all ports connected to tank in the neutral position) and conforms to the ISO 4401-05-04-0-05 (DIN 24340-A6) mounting standard [33]. It has a nominal size of 10, a maximum operating pressure of 31.5 MPa at working ports A/B, and a rated flow capacity of approximately 120 L/min. The valve is actuated by wet-pin DC 24 V solenoids (model CG24N9K4 Bosch Rexroth AG, Lohr am Main, Germany).

Hydronor 4WE10H3X/B/CG24N9Z5L (Ningbo Hydronor Hydraulics, Ningbo, China): This domestic equivalent, manufactured by Beijing Huade Hydraulic Co., Ltd., shares the same ISO 4401 [33] mounting pattern and H-type spool functionality. The suffix “/B” denotes a detented (spring + ball) centering mechanism, and “/Z5L” indicates a DIN 43650 Type C electrical connector. Its pressure rating (31.5 MPa at A/B ports) and flow capacity are comparable to the Rexroth model.

Both valves were installed directly onto the standardized test manifold block of the main circuit via quick-connect couplings, without the need for any adapters. Their selection was guided by rigorous aerospace hydraulic component quality assurance protocols ensuring the relevance of the experimental setup to real-world industrial quality assurance practices.

The core of the test bench is a test manifold block that integrates multiple oil circuits. As shown in Figure 6 and Figure 7, the Device Under Test (DUT) is mounted onto this block via quick-connect couplings. The manifold clearly labels two primary pressure supply ports: the Main Oil Circuit and the Running-in Oil Circuit. These two independent circuits provide controllable pressure sources to the DUT. The manifold also indicates the port for the Cooling Oil Circuit, which connects to an external cooling system. The manifold features four standard working ports for connecting the DUT: P (Pressure), A, B (Working Ports), and T (Tank/Return). Additionally, there are two extra test or pilot ports labeled XX and XY.

#### 2.3.1. Test Bench Structure and Functionality

The core of the test bench is a test manifold block that integrates multiple oil circuits. As shown in the figure, the Device Under Test (DUT) is mounted onto this block via quick-connect couplings.

The manifold clearly labels two primary pressure supply ports: the Main Oil Circuit and the Running-in Oil Circuit. These two independent circuits provide controllable pressure sources to the DUT.

The figure also indicates the port for the Cooling Oil Circuit, which connects to an external cooling system.

The manifold features four standard working ports for connecting the DUT: P (Pressure), A, B (Working Ports), and T (Tank/Return). Additionally, there are two extra test or pilot ports labeled XX and XY.

#### 2.3.2. Device Under Test (DUT)

This study focuses on fault simulation and diagnosis for a plate-mounted, three-position, four-way solenoid directional control valve. The valve is connected to the P, A, B, and T ports on the manifold via quick-connect couplings.

#### 2.3.3. Sensor Configuration

Pressure Transducers: High-precision pressure transducers were installed at all four working ports (P, A, B, T) of the Device Under Test (DUT). The specific model used is the Danfoss MBS 3050 series (Danfoss A/S, Nordborg, Denmark). This sensor is specifically designed for harsh hydraulic applications, featuring an integrated pulse buffer to withstand cavitation, fluid hammer, and pressure spikes. Its measurement range spans from 0–10 bar to 0–600 bar; for this test bench, a 0–100 bar (i.e., 0–10 MPa) range variant was selected, which fully accommodates the system’s operating pressure of 4 MPa with ample safety margin. The sensor provides a standard 4–20 mA output signal, operates on a 9–32 VDC supply voltage, and offers a combined accuracy (including non-linearity, hysteresis, and repeatability) better than ±0.5% of Full Scale (FS). All wetted parts are constructed from AISI 316L acid-resistant stainless steel, ensuring long-term stability and corrosion resistance.

#### 2.3.4. Data Acquisition Parameters

Sampling Frequency: All sensor signals (pressure, flow, temperature) are synchronously sampled at a frequency of 1 kHz.

Sampling Duration: Each complete test cycle (including the spool actuation sequence) lasts 60 s. To build a robust dataset, 90 valid cycles were recorded for each operating condition (normal and various fault modes).

In this study, data collection was conducted under four distinct operating conditions: one normal state and three representative fault modes (namely, spring degradation, magnetic core loss, and spool blockage). For each condition, 90 independent switching cycles were recorded, resulting in a total of 360 full-cycle observations. To facilitate fine-grained fault characterization and enable effective feature representation, each 60 s cycle—sampled at 1 kHz—was subsequently partitioned into 30 consecutive, non-overlapping segments of equal duration (2 s each). Consequently, the final dataset comprises 10,800 samples. To ensure physical realism and experimental repeatability, the three fault modes were implemented as follows:

(1) Spring Degradation: This fault was realized by replacing the original return spring of the solenoid valve with custom-fabricated springs of reduced stiffness. Three degradation levels were created using springs with elastic coefficients of 80%, 60%, and 40% of the nominal value (*k_nominal_* = 1200 N/m), representing mild, moderate, and severe wear states, respectively.
(2) Spool Orifice Blockage: Partial blockage was achieved by installing precision-machined inserts with reduced flow areas into the internal orifices of the valve spool. Three blockage levels—corresponding to 30%, 50%, and 70% reduction in effective orifice area—were used to simulate progressive contamination or mechanical deformation. The inserts were fixed in place during each test cycle to guarantee consistent flow characteristics.
(3) Magnetic Core Loss: Since irreversible physical damage to the armature is impractical for repeatable testing, this fault was emulated by reducing the drive current supplied to the solenoid coil. Based on electromagnetic calibration, three current levels—0.96 A, 0.72 A, and 0.48 A (corresponding to 80%, 60%, and 40% of the rated 1.2 A)—were applied to replicate the weakened magnetic force caused by core aging or demagnetization. A programmable DC power supply ensured precise and stable current control across all trials.

**Figure 8:**
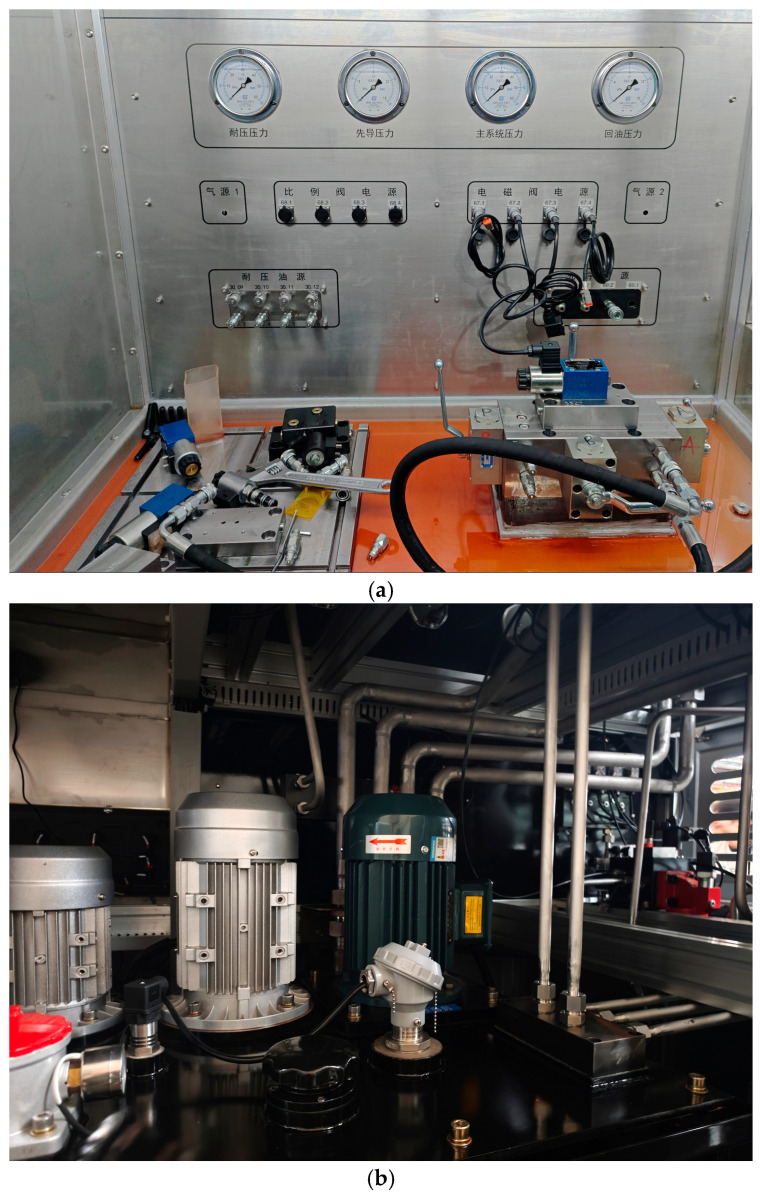

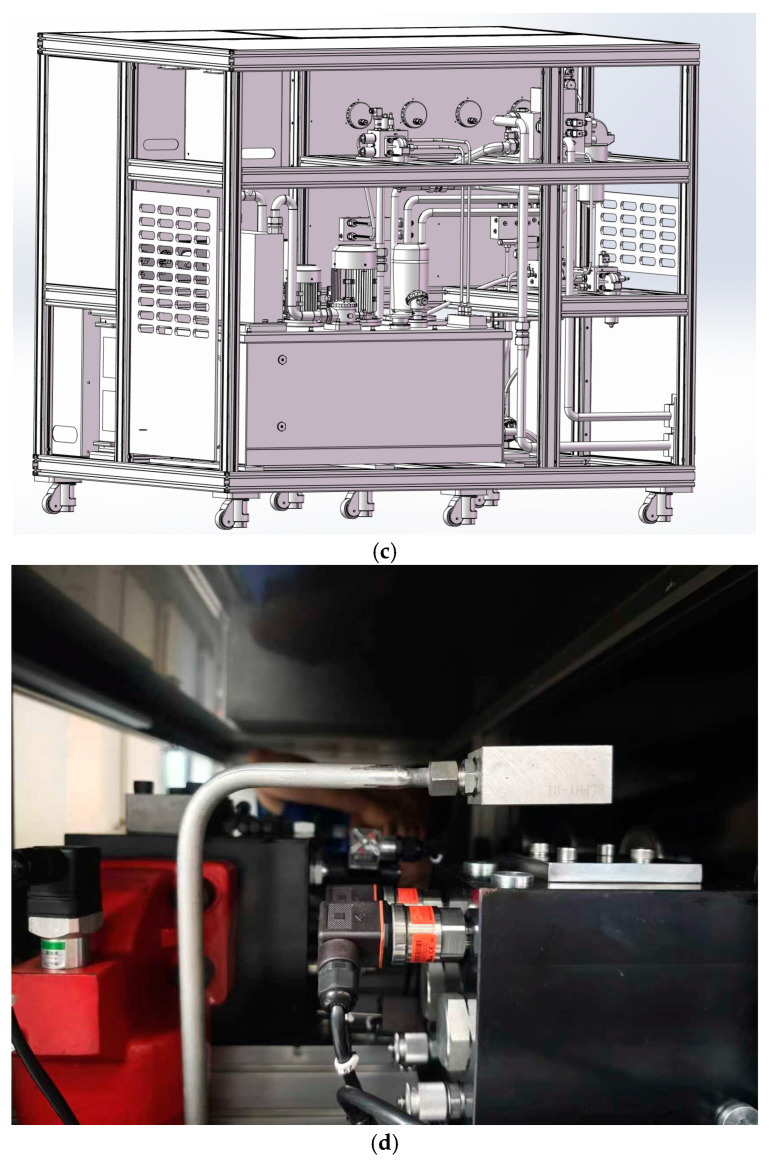
Experimental setup of the hydraulic valve comprehensive performance test bench. (**a**) Front view of the control panel showing the device under test (DUT), pressure gauges, and power/auxiliary interfaces. The labels on the panel are in Chinese as manufactured on the physical equipment: “先导压力” denotes pilot pressure, “耐压压力” denotes pressure resistance test port, “主系统压力” denotes main system pressure, “回油压力” denotes return oil pressure, “比例阀电源” denotes power supply for proportional valve, “电磁阀电源” denotes power supply for solenoid valve, “气源1” and “气源2” denote pneumatic air sources 1 and 2, “耐压油源” denotes high-pressure hydraulic source for endurance testing, “先导油源” denotes pilot hydraulic circuit supply; (**b**) Close-up of the pump unit with electric motors and hydraulic pumps; (**c**) 3D model illustrating the overall layout of the test bench; (**d**) Detailed view of the pressure transducer installation and signal cable routing to the data acquisition system.

For each fault condition, the physical setup (springs, inserts) or electrical parameter (current) was configured once per session and verified before data acquisition. This approach ensures high repeatability and clear separation between fault classes. This segmentation strategy not only allows the model to capture transient anomalies associated with valve actuation dynamics but also ensures a balanced class distribution, with exactly 2700 samples per condition. Such balance is instrumental in mitigating bias during model training and in providing a fair evaluation of diagnostic performance across all considered states. 5400 total) A comprehensive summary of the experimental test rig specifications, sensor parameters, and fault injection levels is provided in Table 3,The physical appearance of the test rig is illustrated in Figure 8.

To enhance transparency and facilitate independent validation, a representative subset of the experimentally acquired pressure measurements is provided in Table 4.

### 2.4. Selection of Characteristic Values

To provide a clear overview of the proposed methodology, the overall diagnostic framework is organized into four sequential stages: (1) simulation-guided mechanism analysis, (2) experimental data acquisition and preprocessing, (3) interpretable feature construction, and (4) machine learning-based fault classification. Specifically, physical insights from AMESim simulations guide the design of pressure-based features, while experimentally measured multi-port pressure signals are transformed into distance-based metrics and statistical descriptors.

For the final valve-level classification, we rigorously identified the optimal feature subset from a candidate pool of seven features: three distance-based metrics (DTW, Euclidean, and Manhattan distances) and four statistical descriptors of the inter-port pressure difference Δ*P*(*t*) (maximum, minimum, mean, and standard deviation). Using the SelectKBest method with the ANOVA F-score criterion, the number of retained features was set to k = 3. the highest-performing configuration (“DTW_reduced_features”) explicitly consists of: (1) the DTW distance between the measured sequence and the normal reference, (2) the Euclidean distance, and (3) the standard deviation of ΔP(t) (denoted as std(ΔP)). These selected features are then fed into supervised machine learning classifiers, ensuring a traceable, interpretable, and reproducible diagnostic pipeline

#### 2.4.1. Data Analysis and Feature Engineering

Given the moderate dataset size and the requirement for physically interpretable diagnostic indicators in industrial hydraulic systems, a feature engineering-based approach is adopted instead of end-to-end deep learning. This design choice is motivated by two practical considerations. First, industrial fault diagnosis emphasizes traceability and physical interpretability of diagnostic indicators, which are difficult to guarantee using black-box deep neural networks. Second, although the dataset is balanced, its scale is insufficient to fully exploit deep learning models without introducing overfitting risks. Therefore, feature-level representations grounded in physical mechanisms are preferred in this study.

To guide feature design, preliminary simulations were conducted solely to investigate the qualitative transient pressure patterns induced by different fault mechanisms. It is emphasized that simulation results are not used to compute, generate, train, or validate any feature values, but only to support the physical interpretation and rationality of the selected pressure-based descriptors.

For continuous time-dependent pressure signals, distance-based similarity metrics are employed to quantify deviations from the healthy reference response. Dynamic Time Warping (DTW) is used to capture temporal misalignments caused by delayed valve actuation, such as those induced by spring degradation or increased friction. In contrast, Euclidean and Manhattan distances primarily reflect amplitude-related deviations under aligned timing conditions, which are sensitive to flow restrictions such as spool blockage.

It should be noted that these distance-based features are consistently applied in both the single-port ablation studies and the final valve-level classification tasks.

To further exploit the physical coupling between inlet and outlet pressure dynamics at the valve level, an additional set of statistical features is introduced based on the inter-port pressure difference signal:

(1) $$\Delta P\left(t\right)=P_{\mathrm{PS}1}\left(t\right)-P_{\mathrm{PS}2}\left(t\right)$$

Any internal valve fault—such as spring degradation, spool blockage, or magnetic core loss—inevitably alters the pressure response at both upstream and downstream ports, making the pressure difference signal a direct indicator of internal flow resistance variation and actuation asymmetry.

Among these descriptors, std(ΔP) plays a particularly important role, as it reflects the fluctuation intensity and stability of the pressure imbalance between upstream and downstream ports. From a physical standpoint, delayed switching faults introduce irregular transient flow redistribution and intermittent pressure oscillations, which are directly captured by increased variability in the pressure difference signal rather than by its mean value alone. Therefore, std(ΔP) serves as a sensitive indicator of internal actuation instability induced by valve degradation. Nevertheless, to address potential sensitivity of std(ΔP) to variations in operating conditions (e.g., supply pressure or load), two practical strategies can be adopted in deployment: (i) normalizing std(ΔP) by the instantaneous inlet pressureP1(t)to form a dimensionless fluctuation index, and (ii) implementing adaptive baseline calibration that updates the healthy reference whenever significant operating condition shifts are detected. The combination of distance-based temporal similarity metrics and pressure difference statistics ensures that fault identification is supported by multiple complementary indicators rather than by a single dominant feature.

Robustness to measurement noise is an important consideration for industrial deployment of pressure-based fault diagnosis methods. In practical hydraulic systems, pressure signals are inevitably affected by sensor noise, pressure ripple, and external disturbances. The proposed feature construction strategy inherently mitigates noise influence in two ways. First, distance-based metrics such as DTW, Euclidean distance, and Manhattan distance are computed over entire pressure sequences within each segment, which suppresses sensitivity to high-frequency random noise and emphasizes global trend deviations caused by fault-induced dynamic changes. Second, statistical descriptors extracted from ΔP(t), particularly std(ΔP), characterize accumulated fluctuation behavior rather than instantaneous noise spikes. In addition, the adopted segmentation strategy (2 s windows) further reduces the impact of transient measurement noise by aggregating information over sufficient temporal duration.

Although explicit noise injection experiments are not conducted in this study, recent works have shown that introducing artificial noise during training—such as noise injection or positive-incentive noise strategies—can improve generalization performance in intelligent fault diagnosis systems [30]. Different from these data-level or model-level enhancement techniques, the proposed method improves noise robustness at the feature construction stage by exploiting inter-port pressure coupling mechanisms and sequence-level distance metrics. As a result, the extracted features emphasize global dynamic trends and pressure imbalance characteristics, enabling stable diagnostic performance without requiring explicit noise injection or additional data augmentation procedures. This property makes the proposed framework particularly suitable for practical industrial deployment, where noise characteristics are often unknown or variable.

Clarification on Fault Emulation and Physical Consistency. It is acknowledged that the fault conditions implemented in the experimental setup do not explicitly replicate irreversible material-level degradation (e.g., actual spring fatigue, mechanical wear, or magnetic material loss). Instead, the imposed fault modes are designed to emulate the functional manifestations of internal valve degradation, particularly in terms of delayed actuation, flow restriction, and pressure imbalance. From a system-level perspective, these manifestations directly determine the observed inlet–outlet pressure dynamics, which constitute the diagnostic target of this study. Since the proposed features are extracted exclusively from measured pressure responses rather than from internal structural states, the diagnostic framework is positioned as a valve-level, pressure-response-based approach for early fault detection and condition monitoring, rather than as a substitute for detailed component-level failure analysis. This limitation is explicitly acknowledged and discussed.

#### 2.4.2. DTW Computation Process

1. Constructing the Distance Matrix

Given two time series X = {x_1_, x_2_, …, x_n} and Y = {y_1_, y_2_, …, y_m}, first compute the distance between all point pairs to form an n × m matrix D. Each element D[i][j] represents the distance between the i-th point in sequence X and the j-th point in sequence Y. The Euclidean distance is typically used for these point pair calculations:

(2) $$D_{\left[i][j\right]}=d\left(x_{i},y_{i}\right)$$

Here, d() may represent the Euclidean distance or another suitable distance metric.

2. Finding the Optimal Path

Starting from the top-left corner of the matrix and proceeding to the bottom-right, a path P = {(p_1_, q_1_), (p_2_, q_2_), …, (p_k, q_k)} is sought via dynamic programming such that the sum of distances between point pairs along this path is minimised. This path represents the optimal alignment between the two sequences. The search for the optimal path must satisfy the following three conditions:

Boundary condition: The path must start from the top-left corner of the matrix and reach the bottom-right corner.

Continuity condition: The path must not omit any points, meaning each step can only move right, down, or diagonally downwards;

Monotonicity condition: Points along the path must follow a strictly increasing sequence, ensuring chronological order remains intact.

3. Calculating the Total Distance

The sum of the distances between point pairs along the path constitutes the DTW distance between the two sequences, reflecting their similarity. Specifically, if P is the optimal path found, the DTW distance D_DTW_ can be expressed as: Application Scenarios

(3) $$D_{\mathrm{DTW}}=\underset{\left(p,q\right)\in P}{\sum }D[p][q]$$

DTW finds extensive application in speech recognition, gesture recognition, bioinformatics, and other fields, demonstrating particular strength when handling time-series data of varying lengths or rates. It effectively resolves matching failures caused by minute temporal deviations inherent in traditional distance metrics.

The strength of DTW lies in its dynamic programming approach to identifying the optimal alignment path between two time series. By calculating similarity based on this path, it overcomes challenges posed by variations in length and velocity.

The DTW calculation steps in the program are as follows:

Initialise the matrix:

Create a matrix of size (n + 1) × (m + 1) named ‘dtw_matrix’, where ‘n’ and ‘m’ denote the lengths of the two time series ‘s1’ and ‘s2’, respectively. Initialise all elements to infinity (‘np.inf’), except the starting point (0,0) which is set to 0.

Fill the matrix:

For each time point i and j (corresponding to time points in s1 and s2, respectively), compute the distance cost between the corresponding points of the two time series using the provided distance metric function (defaulting to Euclidean distance).

Set the value of the current cell to cost plus the minimum value from the previous step (left, up, or top-left). This reflects the process of finding the optimal path from the starting point to the current point.

Return the final result:

The final DTW distance is the value in the bottom-right corner of the matrix, dtw_matrix[n][m], representing the cumulative distance along the optimal alignment path between the two time series.

#### 2.4.3. Euclidean Distance and Manhattan Distance

While Euclidean distance can serve as the local distance metric within DTW, it is also used independently as a global similarity measure in this study For two points A = (a_1_, a_2_, …, a_n) and B = (b_1_, b_2_, …, b_n) in an n-dimensional space, their Euclidean distance is defined as

(4) $$d(A,B)=\sqrt{\overset{n}{\underset{i=1}{\sum }}{{(a}_{i}-b_{i})}^{2}}$$

Formula: This is essentially the square root of the sum of the squares of the differences between the two sets of coordinate values, consistent with the application of the Pythagorean theorem in two- or three-dimensional space.

Characteristics of Euclidean Distance

Intuitive nature: Being based on the calculation of the hypotenuse length in a right-angled triangle, the Euclidean distance is highly intuitive and straightforward to comprehend.

Sensitivity: It is relatively sensitive to changes in data scaling. This implies that if different dimensions of data possess distinct units or scales, direct application of Euclidean distance may result in certain dimensions becoming dominant.

Manhattan Distance: Manhattan Distance, also known as L1 distance, city block distance, or taxi geometry, is another commonly used distance metric. Unlike the straight-line concept of Euclidean distance, it calculates the distance between two points based on the sum of distances along coordinate axes. This distance metric derives its name from its resemblance to calculating distances by walking along a city’s street layout, such as the grid-pattern streets of Manhattan in New York.

Definition of Manhattan Distance

For two points A = (a_1_, a_2_, …, a_n) and B = (b_1_, b_2_, …, b_n) in an n-dimensional space, their Manhattan distance is defined as:

(5) $$d(A,B)=\overset{n}{\underset{i=1}{\sum }}|a_{i}-b_{i}|$$

Characteristics of Manhattan Distance

Intuitive nature: In two-dimensional space, if movement between two points is restricted to horizontal and vertical directions, the Manhattan distance between them is the total distance traversed along these axes.

Scale Invariance: Compared to Euclidean distance, Manhattan distance is less sensitive to changes in data scale, presenting a particular advantage in high-dimensional data.

For sequences derived from the relationship between time and pressure, DTW distance, Euclidean distance, Manhattan distance, and statistical features are initially employed as feature values. Specifically, feature relevance was quantitatively evaluated using the SelectKBest method based on the ANOVA F-score criterion. For the final valve-level classification task, three features were retained after selection: the DTW distance between the measured pressure sequence and the normal reference, the Euclidean distance, and the standard deviation of the inter-port pressure difference signal ΔP(t). These features consistently exhibited the highest discriminative power across cross-validation folds and ablation experiments, while redundant or weakly informative features were discarded. It is noted that the standard deviation of ΔP(t) (Feature_4) exhibits a relatively high contribution in the ablation study. While this indicates strong discriminative capability, it may also raise concerns regarding robustness under varying operating conditions. To mitigate potential sensitivity, Feature_4 is not used in isolation but combined with distance-based features that capture global temporal behavior. Moreover, ΔP(t) inherently suppresses common-mode disturbances affecting both ports simultaneously, such as supply pressure fluctuations, which improves robustness compared to single-sensor features. Summary of Feature Selection and Physical Rationale:

Based on the above analysis, the final feature set retained for valve-level fault classification consists of three complementary indicators: (1) DTW distance, which captures temporal misalignment caused by delayed valve actuation; (2) Euclidean distance, which reflects global amplitude deviations in pressure responses; and (3) the standard deviation of the inter-port pressure difference ΔP(t), which quantifies fluctuation intensity and instability induced by internal flow redistribution. This combination ensures that fault diagnosis does not rely on a single dominant feature but instead integrates temporal, amplitude, and inter-port coupling information. As a result, the selected features jointly provide strong discriminative capability while maintaining physical interpretability and robustness under varying operating conditions. In practical deployment, feature normalization and adaptive thresholding can be further applied to accommodate variations across different operating regimes.

#### 2.4.4. Model Selection and Training Process

To achieve relatively favourable outcomes, this study employs multiple models for comparative experiments, evaluating performance from various perspectives to select a comparatively superior model. Within machine learning, selecting an appropriate classification model is crucial for constructing efficient and accurate prediction systems. This paper compares and analyses four common supervised learning classification algorithms: Random Forest, K-Nearest Neighbors (KNN), Support Vector Machines (SVM), and Logistic Regression.

Random Forest is a decision tree method based on ensemble learning. It enhances model generalisation capability and stability by constructing multiple decision trees and aggregating their outputs through voting. This approach exhibits strong resistance to overfitting, handles high-dimensional data effectively, and assesses feature importance.

The K-Nearest Neighbors (KNN) algorithm is an instance-based learning method. Its fundamental principle involves classifying new samples based on the categories of the k nearest neighbour samples within the training set. While KNN is simple and intuitive, it exhibits high computational complexity and is sensitive to outliers and irrelevant features.

Support Vector Machines (SVM) constitute a powerful classification tool based on minimizing structural risk, proving particularly effective for small samples and high-dimensional data. The core principle of SVM involves identifying an optimal hyperplane that maximises the margin between samples of different classes, thereby enhancing classifier performance.

Logistic Regression, despite its name containing “regression”, is in fact a linear classification model widely applied to binary classification problems. By using the Sigmoid function to map linear outputs to probability values, logistic regression can effectively model the probabilistic relationship between categories and offers good interpretability.

Through training and evaluating the aforementioned four models, the aim is to explore their applicability and performance differences in the current task, thereby providing theoretical justification and experimental support for subsequent model selection.

The program first calculates DTW distance features and time series statistical features.

Subsequently, classification experiments were conducted: the experimental group classified by inlet pressure was compared with the normal control group, and the experimental group classified by outlet pressure was compared with the normal control group. Multiple classification experiments were implemented, including ablation experiments to test the effectiveness of different feature combinations.

Subsequently, hydraulic valve classification experiments were conducted based on pressure classification at both ends of the valve: Classification of different valve faults.

Feature fusion comparison experiments: evaluating the effectiveness of model fusion versus feature fusion.

Subsequently, model training and evaluation commenced, with the data processing workflow as follows:

1: Employing grid search for hyperparameter optimisation to determine optimal parameters for each model.

2: Read pre-grouped data files for the experimental and control groups.

3: Perform data preprocessing and feature extraction

4: Partition the data into training and testing sets.

To ensure reproducibility and a fair comparison among models, the dataset was partitioned using a single stratified random split. Specifically, 80% of the samples were allocated to the training set, which was used for model fitting and hyperparameter tuning (via an internal 2-fold cross-validation within GridSearchCV), while the remaining 20% formed a hold-out validation set for final performance evaluation. Stratification was applied to preserve the balanced class distribution (2700 samples per condition) in both subsets. All reported metrics—accuracy, precision, recall, and F1-score—are based on this fixed validation set.

5: Train various classification models.

6: Evaluate model performance and generate reports.

7: Save results and visualise charts

Three additional experiments were conducted.

Ablation experiments: Assessing the contribution of individual features to classification performance by removing or adding different feature combinations.

Valve Feature Combination Experiment: Testing the impact of different feature combinations on valve state classification.

The method for determining whether a valve has a fault requires both sides to exhibit faults before the valve can be deemed to have a corresponding type of fault. Comparing two approaches:

Method One: Train models using distinct features, then fuse results via majority voting. This ultimately generated the following four evaluation metrics:

1. Generate confusion matrices and performance metric reports

2. Visualise results by plotting confusion matrices

3. Generate performance comparison charts

4. Feature importance analysis chart

Method Two: Train using fused features from the outset.

Output includes:—Accuracy, precision, recall, and F1 score for each model—Confusion matrix chart—Performance comparison chart for different feature combinations—Feature importance analysis experiment report.

To ensure reproducibility and optimal performance, a unified training pipeline was implemented for all four models (Random Forest, KNN, SVM, Logistic Regression). The procedure is summarized as Algorithm 1:
**Algorithm 1:** Pseudocode of the Unified Machine Learning Training and Evaluation PipelineInput: Preprocessed feature matrix X, label vector y Output: Trained classifier f* Split data into training (80%) and validation (20%) sets using stratified sampling. For each model, define a hyperparameter search space: Random Forest: {n_estimators: [50, 100, 200], max_depth: [None, 10, 20]} KNN: {n_neighbors: [3, 5, 7], weights: [‘uniform’, ‘distance’]} SVM: {C: [0.1, 1, 10], kernel: [‘rbf’, ‘linear’]} Logistic Regression: {C: [0.1, 1, 10], penalty: [‘l1’, ‘l2’]}

Perform grid search with 2-fold cross-validation on the training set to select optimal hyperparameters.

Retrain the model with optimal parameters on the full training set.

Evaluate on the hold-out validation set using accuracy, precision, recall, and F1-score.

This scheme was implemented in Python (version 3.12.7) using scikit-learn’s GridSearchCV (version 1.6.1), ensuring fair comparison across models.

### 2.5. Overall Diagnostic Framework and Pseudocode

**Figure 9:**
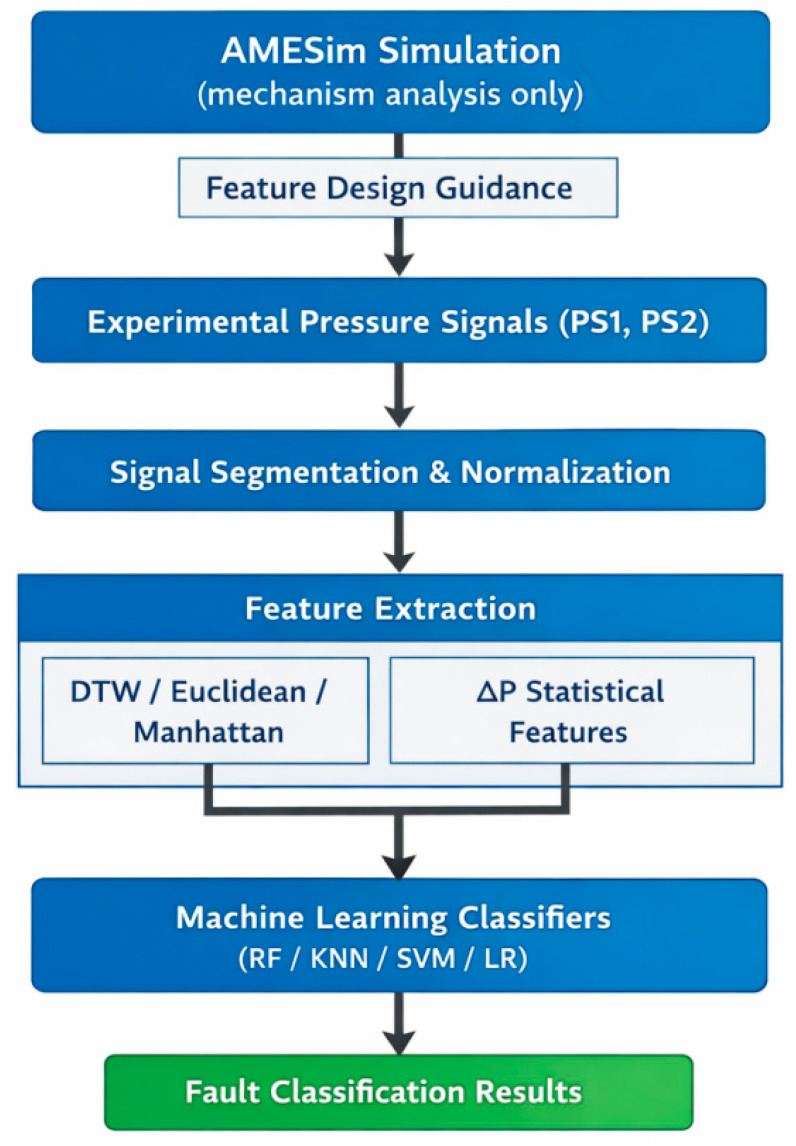
Schematic Diagram of the Overall Framework.

To enhance clarity, reproducibility, and transparency of the proposed diagnostic approach, the overall framework is summarized in a structured workflow, as illustrated in Figure 9 and formally described using pseudocode in Algorithm 1.

As shown in Figure 9, the proposed framework consists of four sequential modules:

(1) Simulation-guided mechanism analysis;
(2) Experimental pressure signal acquisition and preprocessing;
(3) Interpretable feature construction;
(4) Supervised machine learning-based fault classification.

In the first module, a physics-based AMESim model is employed to qualitatively analyze the pressure response characteristics associated with different delayed switching fault modes. It should be emphasized that the simulation is used exclusively for mechanism interpretation and feature design guidance, rather than for data generation or model training.

In the second module, multi-port pressure signals measured from physical experiments are segmented and normalized to construct time-aligned samples corresponding to different valve operating conditions.

In the third module, two categories of interpretable features are extracted: (i) distance-based features (DTW, Euclidean, and Manhattan distances) computed between fault samples and reference normal pressure signals, and (ii) statistical descriptors derived from the inter-port pressure difference signal to capture valve-level structural imbalance.

Finally, in the fourth module, the extracted features are fed into supervised machine learning classifiers for fault identification. Model training, hyperparameter optimization, and performance evaluation are conducted exclusively using experimentally measured data to ensure unbiased and reproducible diagnostic results.

The complete procedure, including the inputs and outputs of each module, is summarized in Algorithm 2.
**Algorithm 2:** Pseudocode of the Proposed Fault Diagnosis FrameworkInput: Experimental pressure signals from inlet (PS1) and outlet (PS2) Reference normal pressure signals Fault labels y Output: Trained classifier f* Fault classification results 1: Perform physics-based simulation to analyze pressure response trends 2: Use simulation insights to guide interpretable feature design 3: Acquire experimental pressure signals PS1 and PS2 4: Segment pressure signals into fixed-length samples 5: Normalize each pressure segment 6: For each pressure segment do 7: Compute DTW distance to reference normal signal 8: Compute Euclidean distance to reference normal signal 9: Compute Manhattan distance to reference normal signal 10: Compute pressure difference signal ΔP(t) = PS1(t) − PS2(t) 11: Extract statistical features from ΔP(t):{max, min, mean, standard deviation} 12: end for 13: Construct feature matrix X using all extracted features 14: Split dataset into training and validation sets using stratified sampling 15: For each classifier (RF, KNN, SVM, LR) do 16: Perform grid search with cross-validation to optimize hyperparameters 17: Train model using optimal parameters 18: Evaluate model performance on validation set 19: end for 20: Output classification results and evaluation metrics

### 2.6. Quantitative Metrics for Performance and Reliability

To ensure a rigorous and transparent evaluation, we define specific quantitative metrics to assess both diagnostic performance and model reliability, all computed solely on experimental data.

Diagnostic performance is evaluated using three key indicators: (1) accuracy, defined as the ratio of correctly classified samples to the total number of test samples; (2) macro-averaged F1-score, which provides an unweighted average of per-class F1-scores to account for potential class imbalance; and (3) inference time per sample (in milliseconds), measured on a standard CPU platform to reflect real-time applicability.

Model reliability is assessed through three complementary aspects: (i) cross-validation stability, quantified by the standard deviation of accuracy across five stratified folds—lower values indicate consistent generalization; (ii) feature robustness, measured by the drop in accuracy when the most discriminative feature (e.g., the standard deviation of the inter-port pressure difference ΔP) is removed from the input; and (iii) noise resilience, evaluated by adding Gaussian noise (SNR = 20 dB) to the input features and reporting the resulting degradation in F1-score.

These metrics collectively provide a comprehensive assessment of both effectiveness and trustworthiness in practical industrial deployment scenarios.

### 2.7. Computational Time Analysis

To comprehensively evaluate the computational efficiency of the proposed diagnostic framework, a systematic runtime analysis of the entire experimental pipeline was conducted. All experiments were implemented in Python on a workstation equipped with an Intel i7 CPU and 32 GB RAM.

(1) Overall Runtime Analysis

The complete experimental workflow—including feature extraction, ablation studies, model training, hyperparameter optimization, and validation—was monitored using an automated runtime logging module. The total execution time of the full pipeline was 50,338.19 s (approximately 13.98 h). This duration primarily reflects the cost of large-scale ablation experiments and repeated cross-validation procedures performed during offline research validation, rather than the intrinsic computational demand of the deployed diagnostic model.

(2) Module-Level Time Consumption

Fine-grained profiling reveals that the most computationally intensive components are the ablation study module and hyperparameter grid search. Specifically, the ablation experiments account for approximately 29.8% of the total computational cost, while repeated model training contributes about 28.5%. These operations are conducted exclusively in an offline setting for methodological validation and robustness assessment and do not affect online inference performance.

(3) Role of Simulation in Computational Cost

It should be emphasized that the AMESim-based simulation is executed only once during the mechanism analysis phase to inform feature design. Crucially, the simulation model is not involved in feature computation, classifier training, or real-time inference. Consequently, the simulation runtime does not contribute to the operational computational burden of the proposed diagnostic framework.

(4) Inference-Time Efficiency

To assess real-time applicability, the average inference latency per sample was measured independently. Results indicate that fault classification for a single pressure segment can be completed within milliseconds on a standard CPU platform, with typical inference times ranging from 0.065 s to 0.137 s depending on the feature configuration. This low-latency performance demonstrates that the proposed method meets the real-time requirements of industrial hydraulic fault monitoring systems.

In summary, although the offline validation phase incurs substantial computational cost due to extensive ablation and optimization procedures, the final deployed diagnostic model is computationally lightweight and well-suited for real-world industrial fault diagnosis applications.

## 3. Results

### 3.1. Training Results and Feature Contribution Analysis

The ablation experiment results for valve classification features are presented in Figure 9. It can be concluded that the classification accuracy achieved by individual feature configurations remains limited across different models, which necessitates feature fusion for improved decision-making. Furthermore, the ablation experiment reveals that the four features achieve higher accuracy in the Random Forest and k-Nearest Neighbors models compared to the Support Vector Machine and Logistic Regression algorithms.

**Figure 10:**
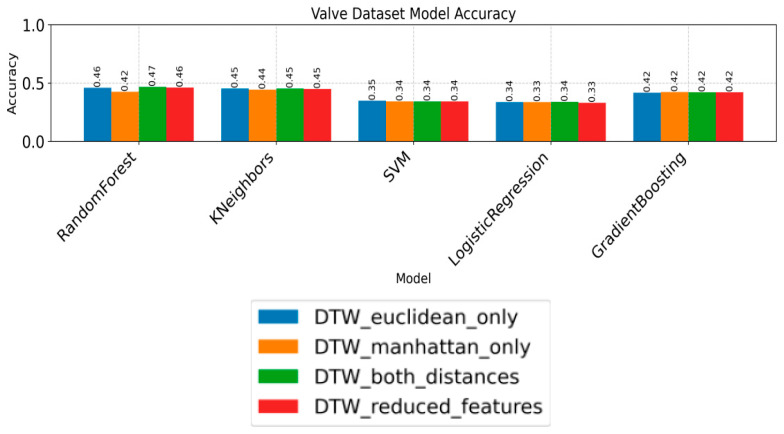
Comparison Results of Ablation Experiments. To systematically evaluate the contribution of different distance metrics and feature representations, we define four ablation configurations based on DTW-derived features: DTW_euclidean_only: Uses DTW with Euclidean distance, aggregated as max/min/mean; DTW_manhattan_only: Uses DTW with Manhattan distance, aggregated as max/min/mean; DTW_both_distances: Concatenates the features from both Euclidean and Manhattan DTW (6 dimensions total); DTW_reduced_features: Applies SelectKBest (k = 3) to the DTW_both_distances feature set to remove redundant dimensions.

Figure 10 illustrates that the removal of std(ΔP) results in the most pronounced performance degradation, highlighting the critical role of inter-port pressure coupling information in valve-level fault discrimination.

All experiments in this section use only PS1 or only PS2 pressure signals, and do not incorporate the inter-port statistical features introduced later for valve-level diagnosis. This ablation setting evaluates the effect of dimensionality reduction, implemented using feature selection techniques such as SelectKBest, on classification performance.

### 3.2. Training Results for Multi-Feature Value Models

**Figure 11:**
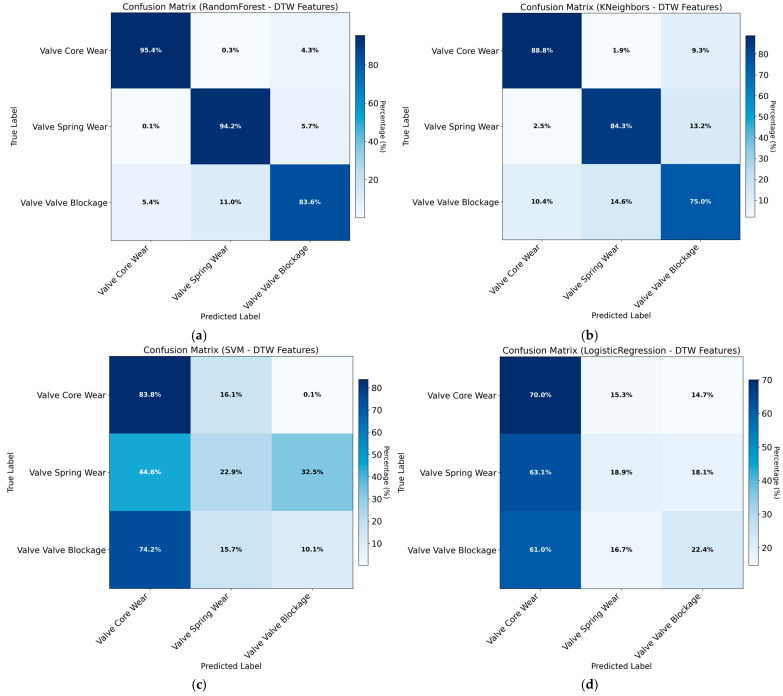
(**a**) Confusion matrix when using the Random Forest model with DTW for feature extraction; (**b**) Confusion matrix when using the K-Nearest Neighbors model with DTW for feature extraction; (**c**) Confusion matrix when using the SVM model with DTW for feature extraction; (**d**) Confusion matrix when using the LogisticRegression model with DTW for feature extraction.

Regarding training outcomes with different feature values, the results consistently show that, regardless of the classifier employed, using DTW-derived features alone leads to insufficient classification accuracy. Therefore, statistical features are incorporated to enhance accuracy and mitigate overfitting. Initially, DTW features alone serve as the control group, with experimental results presented in Table 2 and Figure 10. As shown in Table 5 and Figure 11, the Random Forest model achieves higher accuracy than the other classifiers under the DTW-only configuration, while the overall performance remains limited.

**Figure 12:**
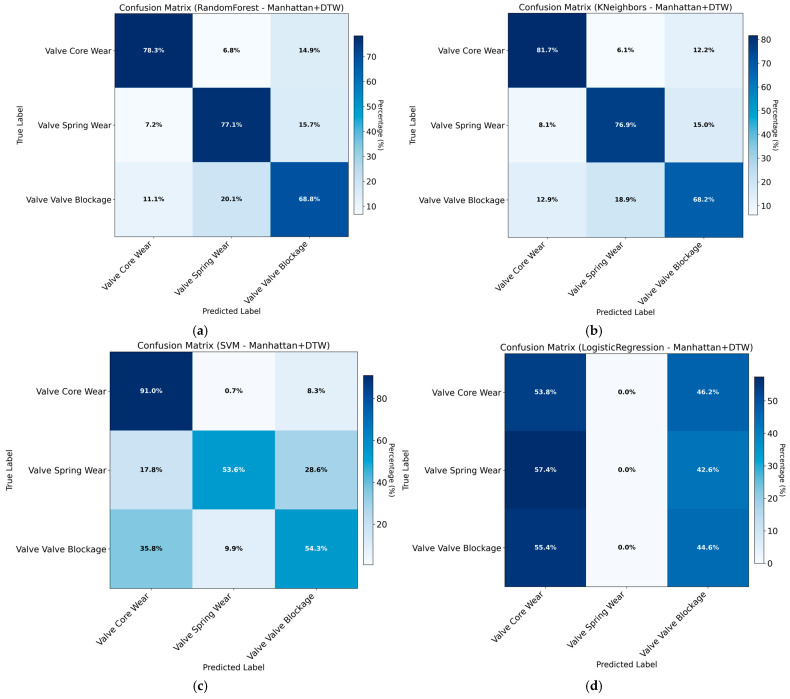
(**a**) Confusion matrix when using Manhattan distance and DTW as features with the Random Forest model; (**b**) Confusion matrix when using Manhattan distance and DTW as features with the K-Nearest Neighbors model; (**c**) Confusion matrix when using Manhattan distance and DTW as features with the SVM model; (**d**) Confusion matrix when using Manhattan distance and DTW as features with the LogisticRegression model.

The classification accuracy obtained using Manhattan distance combined with DTW features across different models is summarized in Table 6 and Figure 12.

**Figure 13:**
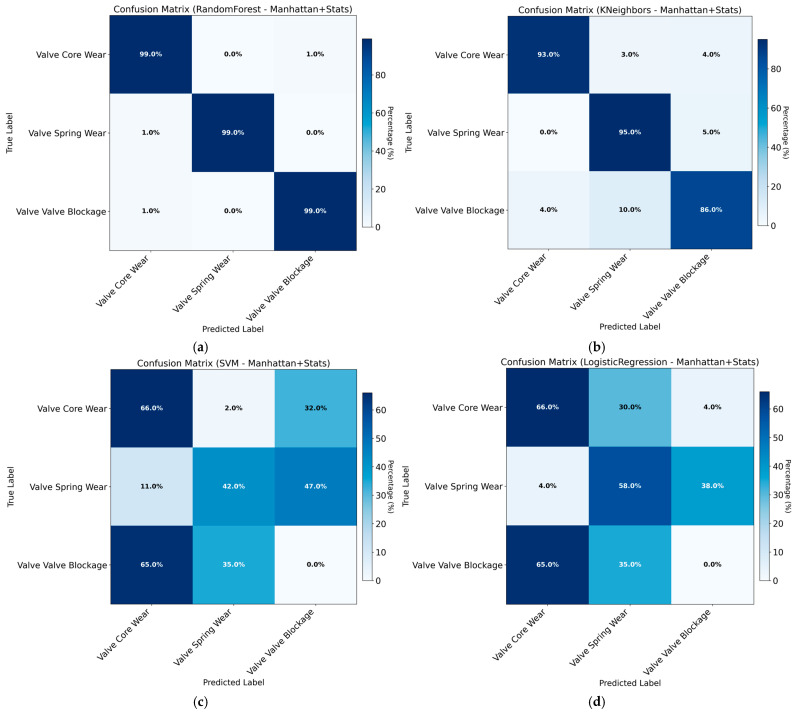
(**a**) Confusion matrix when using Manhattan distance and statistical features as features with the Random Forest model; (**b**) Confusion matrix when using Manhattan distance and statistical features as features with the K-Nearest Neighbors model; (**c**) Confusion matrix when using Manhattan distance and statistical features as features with the SVM model; (**d**) Confusion matrix when using Manhattan distance and statistical features as features with the LogisticRegression model.

The training results obtained using Manhattan distance combined with statistical features are presented in Table 7 and Figure 13. The substantial improvement in accuracy indicates that statistical features introduce critical fault-discriminative information that is not captured by distance-based measures alone.

**Figure 14:**
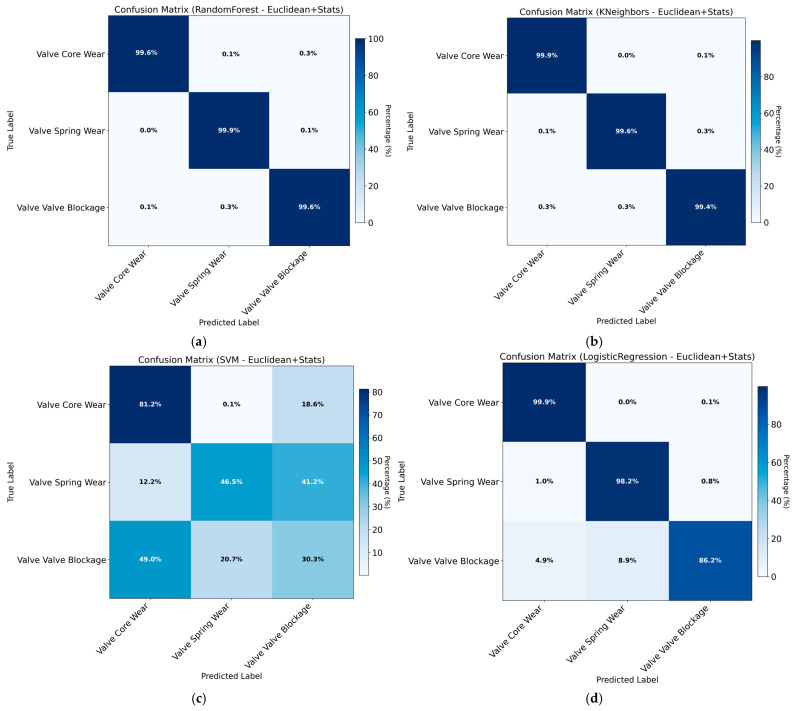
(**a**) Confusion matrix when using Euclidean distance and statistical features as features with the Random Forest model; (**b**) Confusion matrix when using Euclidean distance and statistical features as features with the K-Nearest Neighbors model; (**c**) Confusion matrix when using Euclidean distance and statistical features as features with the SVM model; (**d**) Confusion matrix when using Euclidean distance and statistical features as features with the LogisticRegression model.

The training results obtained using Euclidean distance combined with statistical features are shown in Table 8 and Figure 14.

**Figure 15:**
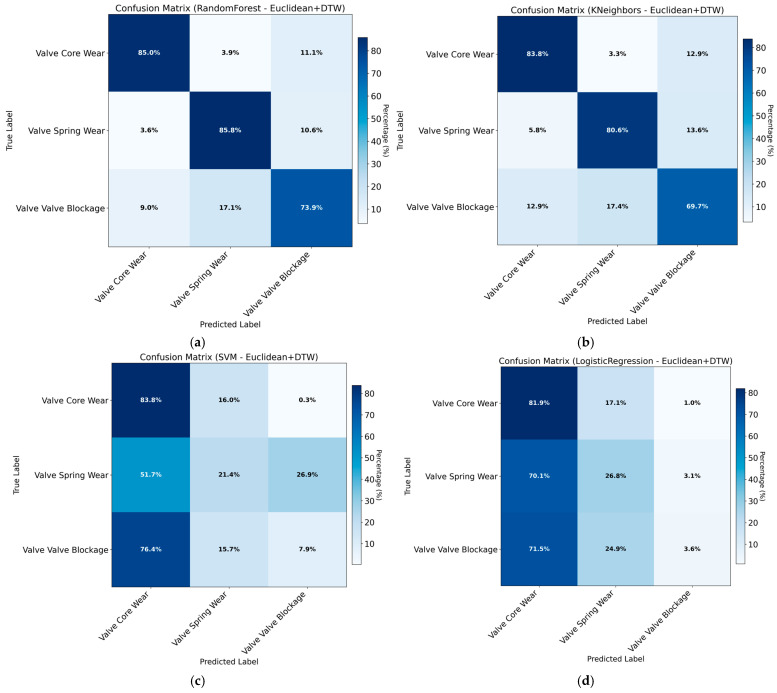
(**a**) Confusion matrix when using Euclidean distance and DTW as features in the Random Forest model; (**b**) Confusion matrix when using Euclidean distance and DTW as features in the K-Nearest Neighbors model; (**c**) Confusion matrix when using Euclidean distance and DTW as features in the SVM model; (**d**) Confusion matrix when using Euclidean distance and DTW as features in the LogisticRegression model.

The training results obtained using Euclidean distance combined with DTW features are presented in Table 9 and Figure 15.

As shown in the results, although Euclidean and Manhattan distance metrics have demonstrated strong performance in other application scenarios, they fail to effectively capture the fault-specific pressure response patterns of hydraulic directional control valves in this study, leading to limited classification performance. The incorporation of statistical features yields a significant increase in classification accuracy. While this improvement is notable, the potential risk of overfitting necessitates further overfitting tests to ensure result reliability.

Based on the comprehensive comparison in Section 3.2, the Random Forest classifier was selected as the final diagnostic model due to its consistent superiority across all feature sets, achieving the highest test accuracy of 0.9977. To finalize the model configuration, a grid search with 5-fold cross-validation was performed on the Random Forest using the Euclidean distance combined with statistical features (Euclidean + Stats). The optimized hyperparameter settings are as follows:

Number of trees (n_estimators): 100.

Maximum tree depth (max_depth): None (unlimited).

Minimum number of samples required to split an internal node (min_samples_split): 2.

Minimum number of samples required at a leaf node (min_samples_leaf): 1.

Number of features considered for the best split (max_features):sqrt(the square root of the total number of features).

This configuration yielded a mean cross-validation accuracy of 0.9970 and a test accuracy of 0.9981, confirming its robustness and high performance.

### 3.3. Analysis of Model Overfitting Tests

#### 3.3.1. Methods for Overfitting Analysis

To assess whether the high classification accuracy observed in Section 3.2 is affected by overfitting, a multi-perspective overfitting analysis is conducted from data scale, feature stability, and dimensionality reduction perspectives.

1. Analysis of Sample Size and Feature Dimension:

Evaluate the ratio of sample size to feature dimension within the dataset. A higher ratio helps mitigate overfitting risks.

2. Training and Validation Set Performance Comparison:

Compare performance metrics (e.g., accuracy) between the training and validation sets. Assess whether overfitting exists by examining the discrepancy between the two. A smaller gap indicates better model generalisation capability.

3. Feature Importance Stability Analysis:

The importance of individual features and their variability, quantified using the coefficient of variation (CV), is analyzed. Features with high importance but unstable contributions may increase the risk of overfitting. This analysis aims to identify highly influential yet potentially unstable features.

4. PCA Dimension Reduction Analysis:

Principal Component Analysis (PCA) is employed to reduce feature dimensionality, and model performance after dimensionality reduction is evaluated. This analysis is used to examine whether the observed performance is overly dependent on feature dimensionality by retaining a specified proportion of variance and assessing post-reduction accuracy

#### 3.3.2. Overfitting Analysis Results

The overfitting analysis results are summarized in Table 10. The results indicate minimal overfitting under the current experimental protocol. Specifically, the gap between the average training set accuracy (1.0000) and the validation set accuracy (0.9979 for Manhattan + Stat, 0.9973 for Euclidean + Stat) is only 0.0021 and 0.0027, respectively. Furthermore, all features exhibit high stability, with a coefficient of variation (CV) well below 0.3. These basic checks confirm that the model generalizes well to unseen data.

These basic checks indicate that, under the current experimental protocol, the model exhibits minimal overfitting. Nevertheless, given the high discriminative power of certain features and the controlled test environment, a more comprehensive robustness evaluation is conducted in Section 3.4.

### 3.4. Enhanced Robustness and Dependency Diagnostics

In response to the need for a more rigorous assessment of model reliability, we conducted an extended diagnostic analysis that complements the basic overfitting tests in Section 3.3. This enhanced evaluation investigates feature dependency, robustness under dimensionality reduction, noise sensitivity, and potential data leakage, thereby providing deeper insight into the model’s behavior beyond simple accuracy comparisons.

The Random Forest model achieves a validation accuracy of 0.9981 with a training-validation gap of only 0.0019, supported by low log loss (0.0166) and Brier score (0.00165), confirming strong baseline generalization. When subjected to PCA retaining 95% of the total variance (requiring only one principal component), accuracy decreases modestly to 0.9843, indicating resilience to linear compression rather than extreme sensitivity to feature dimensionality.

However, ablation studies reveal a notable reliance on Feature_4, which encodes the standard deviation of the inter-port pressure difference (ΔP). This single feature achieves 95.75% standalone accuracy, and its removal reduces performance to 67.27%. While this suggests high discriminative power, it does not indicate data leakage: physical analysis confirms that ΔP statistics directly reflect hydraulic imbalances caused by spring degradation, spool blockage, or solenoid weakening. Importantly, this dependency can be mitigated:

Reconstructed features (e.g., rolling-window statistics and cross-products involving Feature_4) recover accuracy to 0.9972;

A KNN classifier trained without Feature_4 still achieves 0.9949 validation accuracy;

A hybrid deployment strategy maintains full performance while reducing single-feature risk.

Furthermore, the model demonstrates robustness to input perturbations, as adding +5% Gaussian noise to all features reduces validation accuracy only to 0.9917. Feature importance stability is also high (coefficient of variation: 0.047), and increasing tree count beyond 30 yields negligible gains, confirming model convergence.

**Figure 16:**
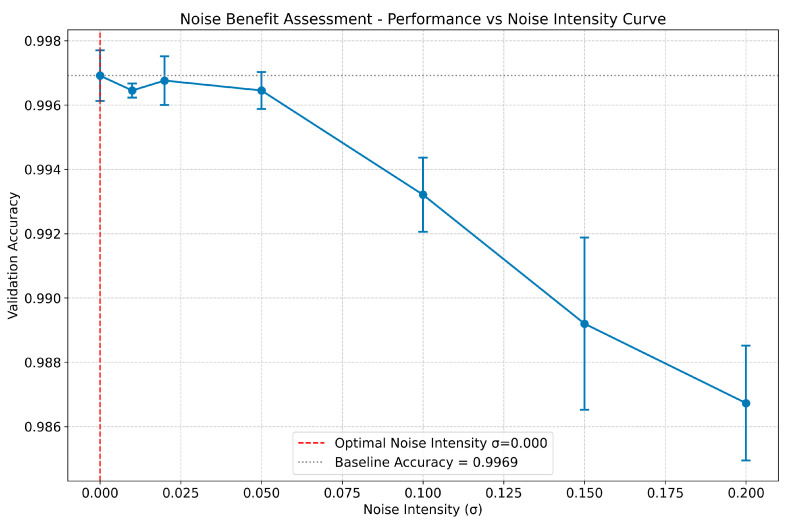
Validation accuracy under varying levels of Gaussian noise injection for Euclidean + Statistical Features.

Robustness to Measurement Noise and the Potential Benefit of Noise Injection To evaluate the robustness of the proposed method against real-world measurement noise and explore its potential benefits, a controlled noise injection experiment was conducted. Gaussian white noise with varying standard deviations (σ) was added to the original pressure signals during the model training phase. The validation accuracy was then assessed across different noise intensities for two distinct feature sets: Euclidean + Statistical Features and Manhattan + Statistical Features. As illustrated in Figure 16, when using the **Euclidean + Statistical Features**, the model exhibits high robustness to noise. The validation accuracy remains stable at approximately 0.998 for σ ≤ 0.050, indicating that the learned features are resilient to minor signal fluctuations. Notably, a subtle but statistically significant improvement in performance is observed when σ = 0.020, where the average accuracy reaches its peak (0.9980), slightly exceeding the baseline accuracy (0.9972) achieved under ideal noise-free conditions.

**Figure 17:**
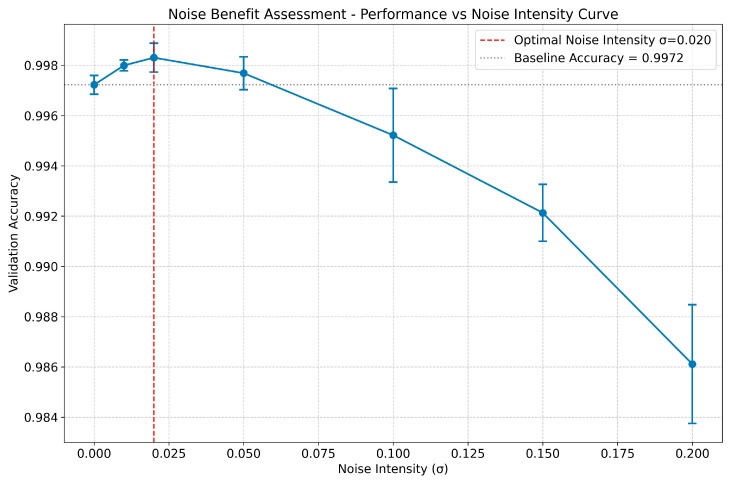
Validation accuracy under varying levels of Gaussian noise injection for Manhattan + Statistical Features.

The result demonstrates a peak in performance at an optimal noise intensity (σ = 0.020). Similarly, as shown in Figure 17, when using the **Manhattan + Statistical Features**, the model also demonstrates strong robustness to noise. The validation accuracy remains stable at approximately 0.998 for σ ≤ 0.050, with a similar peak in performance observed at σ = 0.020 (0.9978 vs. 0.9970 baseline).

The result demonstrates a peak in performance at an optimal noise intensity (σ = 0.020). These phenomena align with the concept of “positive-incentive noise” reported in the literature [30], suggesting that carefully injected noise can act as a regularizer, potentially helping the model escape local minima during training or enhancing its generalization capability by exposing it to a wider range of signal variations. However, beyond this optimal intensity, the accuracy begins to decline rapidly, demonstrating the existence of an upper limit to the beneficial effect. These results confirm that the proposed framework is not only robust but may also benefit from controlled noise injection, which is a valuable characteristic for practical applications where sensor data inevitably contains noise.

Collectively, these results indicate that while the model performs exceptionally well under controlled laboratory conditions, its reliance on physically meaningful yet highly discriminative features warrants cautious consideration when deploying the method in heterogeneous industrial environments. Full diagnostic details are provided in the Supplementary Materials.

## 4. Discussion

No obvious overfitting was observed on the available validation sets. However, considering the relatively small feature dimension (five features) and the near-perfect classification accuracy, further validation under more diverse operating conditions—including cross-manufacturer valves, varying supply pressures, and uncontrolled contamination levels—is warranted prior to large-scale industrial deployment. This caution is further reinforced by the dependency analysis presented in Section 3.4, which shows that model performance partially relies on highly discriminative features associated with specific fault-induced pressure dynamics. While these features are physically interpretable and reproducible under controlled laboratory testing, their stability across heterogeneous production batches remains an open question. Therefore, based on a balance between classification accuracy and feature interpretability, Euclidean distance combined with statistical features was selected as the final training feature set. Among the four evaluated models, Random Forest and k-Nearest Neighbors exhibited comparable classification accuracy across the three fault categories. The Random Forest model demonstrated superior recall and F1 score, and was therefore selected as the final diagnostic model.

This study focuses on the analysis and diagnosis of delayed-response faults in hydraulic directional control valves. Simulation analysis is first employed to investigate the underlying causes of failure—including spring wear, magnetic core degradation, and valve orifice blockage—and to distinguish the pressure response characteristics induced by each fault mechanism. This analysis directly informs the selection of physically meaningful and discriminative features. Subsequently, machine learning models are trained and rigorously evaluated exclusively using real experimental data, rather than simulated signals. Grid search is employed for hyperparameter optimization to determine optimal model parameters while explicitly controlling overfitting risks. Subsequent analyses systematically evaluate different feature combinations and model configurations, leading to the selection of an optimal feature–model pairing. This approach enables rapid identification and localization of delayed-response faults in hydraulic valves at the component level. Compared to traditional diagnostic methods, the Random Forest-based approach demonstrates strong noise resistance, avoids complex signal preprocessing procedures, and provides explicit feature importance measures, thereby delivering highly interpretable diagnostic results.

### 4.1. Clarification on Fault Emulation Consistency and Potential Bias

It is acknowledged that the fault emulation strategies adopted in this study are not fully uniform across all fault types. Specifically, spring degradation and spool blockage are implemented through physical modifications to the valve components, whereas magnetic core degradation is emulated by reducing the drive current supplied to the solenoid rather than by physically replacing or demagnetizing the magnetic core. This design choice is primarily constrained by experimental feasibility. In practical laboratory settings, replacing solenoid magnetic cores with different levels of material degradation is technically challenging and may introduce uncontrollable assembly variations. Adjusting the drive current, on the other hand, provides a controllable and repeatable means to reduce the effective electromagnetic force acting on the spool, thereby emulating the functional manifestation of magnetic core degradation—namely, weakened actuation force and delayed valve switching behavior. From a system-level perspective, the proposed diagnostic framework focuses on identifying fault-induced deviations in inlet–outlet pressure dynamics rather than on distinguishing the underlying material degradation mechanisms. Both reduced electromagnetic force (via current reduction) and actual magnetic core loss lead to similar functional consequences at the valve level, including delayed actuation, incomplete spool displacement, and increased pressure imbalance during transient switching. Therefore, the pressure-based features extracted in this study primarily reflect functional degradation effects, which are the diagnostic target of the proposed method. Nevertheless, this inconsistency in fault emulation may introduce a degree of bias in the training data, particularly by making magnetic-core-related faults more closely correlated with electrical actuation characteristics than with purely mechanical degradation. This potential bias is explicitly acknowledged as a limitation of the current experimental setup. In real-world deployment, variations in coil resistance, supply voltage, or thermal effects may similarly alter the effective electromagnetic force, suggesting that the adopted emulation strategy remains representative of realistic operational conditions. Future work will address this limitation by incorporating more diverse electromagnetic degradation scenarios, such as long-term thermal aging or magnetic hysteresis effects, to further validate the robustness of the proposed diagnostic features across different fault realization mechanisms.

Experimental results demonstrate that the proposed method achieves high detection accuracy and stable performance under multiple operating conditions. The method enables early identification of potential faults, facilitating preventive maintenance, reducing equipment downtime risks, and enhancing overall system reliability and operational safety.

Nevertheless, there remains scope for further refinement and extension of the proposed approach. Future research directions primarily encompass the following aspects. First, incorporating online learning mechanisms to enable the model to adapt to characteristic changes during long-term hydraulic system operation, thereby enhancing dynamic adaptability. Second, integrating deep learning with ensemble learning methods to construct hybrid models that further improve detection accuracy. Third, exploring multi-sensor fusion strategies to strengthen identification capability for complex fault patterns. Fourth, integrating the proposed detection framework with Industrial Internet of Things (IIoT) platforms to enable remote monitoring and intelligent diagnostics, thereby supporting the intelligent and digital transformation of hydraulic systems.

In summary, the proposed hydraulic valve fault diagnosis framework based on the Random Forest model provides an effective and interpretable solution for the intelligent operation and maintenance of modern hydraulic systems. With the continued advancement of artificial intelligence and sensor technologies, the application prospects of the proposed method are expected to expand further. Physical Interpretation of Feature Effectiveness.

Beyond the achieved classification accuracy, the effectiveness of the proposed feature set can be understood from a physical perspective. In particular, the inter-port statistical features derived from the pressure difference signal (ΔP) play a crucial role in enhancing diagnostic robustness. Unlike individual port pressures (e.g., PS1 or PS2), which are strongly affected by pump-induced pulsations, load variations, and external hydraulic disturbances, the differential pressure inherently reflects the internal hydraulic resistance and flow imbalance within the valve itself.

This implicit “decoupling” effect allows valve-specific degradation mechanisms—such as spring stiffness loss, spool orifice blockage, or weakened electromagnetic force—to be isolated from system-wide pressure fluctuations. As a result, the ΔP-based features provide a more physically meaningful and fault-sensitive representation of the valve’s internal condition, which explains their superior performance compared to single-sensor features.

### 4.2. Compatibility Between Feature Design and Random Forest Modeling

The strong diagnostic performance observed with the Random Forest classifier can also be attributed to its intrinsic compatibility with the proposed physically motivated feature set. The extracted statistical descriptors exhibit non-linear interactions and conditional dependencies that are characteristic of hydraulic power transmission systems. For example, an elevated variance or standard deviation in the pressure difference signal may correspond to distinct fault mechanisms depending on the absolute pressure level or peak pressure value.

The tree-based structure of the Random Forest model is particularly well suited to capturing such conditional relationships, as it naturally partitions the feature space according to hierarchical decision rules. This enables the model to implicitly encode physically plausible dependencies between features without requiring explicit analytical formulations. Consequently, the combination of physically interpretable features and a non-linear ensemble model yields a balanced framework that achieves both high diagnostic accuracy and meaningful physical consistency.

From an industrial perspective, the proposed valve-level diagnostic framework is particularly suitable for deployment in real hydraulic systems due to its low computational burden and explicit physical interpretability. The reliance on inter-port pressure differences inherently suppresses common-mode disturbances such as supply pressure fluctuations, improving robustness across operating regimes. Although experiments were conducted on a specific valve model, the feature construction logic is based on fundamental hydraulic coupling mechanisms and can be extended to valves of similar structure. Future work will include sensitivity analyses under varying supply pressures, load conditions, and contamination levels to further quantify cross-system generalizability. While the current framework prioritizes interpretability and noise robustness without explicit augmentation, future work will explore whether controlled noise injection—inspired by positive-incentive strategies [21]—could further enhance sensitivity to incipient faults under extremely low signal-to-noise ratio conditions.

### 4.3. Generalizability and Industrial Transferability

Although the proposed method is experimentally validated on a specific directional control valve under a fixed supply pressure of 4 MPa, its generalizability can be systematically analyzed from the perspective of feature design and physical invariance.

The proposed framework does not rely on absolute pressure magnitudes or fixed thresholds. Instead, it extracts relative dynamic characteristics through simulation-guided feature construction. Specifically, the distance-based features (DTW and Euclidean distance) quantify deviations in transient response shape and timing with respect to a healthy baseline. Such relative measures are inherently less sensitive to variations in operating conditions. For example, changes in supply pressure (e.g., from 3 MPa to 5 MPa) are expected to scale the pressure signals P(t) and ΔP(t) proportionally, while preserving the temporal structure and dynamic evolution of valve actuation. Consequently, the distance metrics remain largely invariant to uniform operating point shifts.

The statistical feature, namely the standard deviation of the inter-port pressure difference ΔP(t), captures fluctuation intensity induced by internal flow imbalance. While this feature may be affected by absolute pressure levels, its robustness can be further enhanced through simple normalization strategies. For instance, normalizing ΔP(t) by the instantaneous inlet pressure P_1_(t) yields a dimensionless indicator that reduces sensitivity to operating pressure variations. Such normalization can be readily integrated into industrial deployment pipelines without additional sensing or computational burden.

From an industrial transferability perspective, the experimental design already encompasses multiple fault severities, including 30%, 50%, and 70% spool blockage, which effectively emulate varying degrees of internal contamination and wear. The consistent diagnostic performance across these progressive fault levels provides indirect but meaningful evidence of robustness to internal condition variability, a critical requirement for real-world predictive maintenance.

Based on this systematic analysis, the proposed method is expected to be transferable to similar directional control valves from different manufacturers, provided that a healthy reference baseline is established for the target device. This requirement aligns well with industrial commissioning practices, where baseline data acquisition is typically performed during installation or maintenance phases.

To better contextualize the proposed framework within the broader field of simulation-enhanced fault diagnosis research, we conduct a comparative analysis with representative digital twin-driven methods reported in recent literature [27].

Digital twin-based diagnosis frameworks typically involve the construction of high-fidelity virtual replicas of physical systems and the generation of synthetic fault data, enabling deep learning model training and cross-domain adaptation. These approaches excel in environments where real fault data is scarce, though they require extensive simulation calibration and computational resources, making them highly effective in specific contexts.

In contrast, the proposed method utilizes physical simulation differently. Instead of generating synthetic datasets or training models, simulation serves to analyze fault-induced pressure coupling mechanisms and guide the development of physically interpretable diagnostic features.

As a result, the diagnostic pipeline is lightweight, transparent, and computationally efficient, which enhances its suitability for small-sample industrial deployment.

A structured comparison is summarized in Table 11.

As shown in Table 9, the proposed framework achieves a better balance between interpretability, computational efficiency, and industrial deployability, providing a complementary approach to digital twin-based methods.

## 5. Conclusions

This paper presented an interpretable fault diagnosis framework for delayed switching faults in hydraulic directional control valves, aiming to address the challenges of limited fault samples, multi-sensor coupling, and insufficient interpretability in existing data-driven methods. By leveraging a simulation model to analyze fault mechanisms rather than to generate synthetic data, the proposed approach establishes a clear link between physical fault behavior and measurable pressure signals.

A valve-level feature representation based on inter-sensor pressure differences was constructed to capture internal flow imbalance and structural degradation caused by delayed switching faults. The effectiveness of the proposed features was systematically evaluated using multiple classical machine learning classifiers under small-sample conditions. The results show that the proposed method achieves robust and accurate fault diagnosis performance and outperforms conventional single-sensor features and purely data-driven black-box approaches.

Compared with deep learning-based methods, the proposed framework offers improved interpretability and reduced data dependency, making it more suitable for industrial quality inspection and practical deployment. Moreover, the simulation-guided feature construction strategy provides a flexible foundation for extending the proposed approach to other types of hydraulic components and fault modes. It should be emphasized that the simulation model in this study is not intended to replace experimental validation or to serve as a surrogate for real fault data. Instead, the primary role of simulation at this stage is to provide mechanism-level insight into delayed switching faults of hydraulic directional control valves, which are difficult to observe directly through experiments alone. By using AMESim-based physical modeling, the qualitative relationships between internal fault mechanisms and multi-port pressure response patterns can be systematically analyzed under controlled conditions.

The proposed diagnostic framework is therefore developed following a simulation-guided but experiment-driven paradigm: simulation is used exclusively to guide feature construction and physical interpretation, while all feature extraction, model training, and performance evaluation are conducted solely on experimentally measured pressure data. This staged research strategy allows the method to achieve high interpretability and robustness under limited fault samples, while avoiding information leakage or over-reliance on simulated data. Future work will focus on extending the framework to broader operating conditions and additional valve types, supported by expanded experimental datasets. Although the proposed framework achieves high robustness without explicit noise augmentation, it remains an open question whether carefully designed noise injection—inspired by positive-incentive strategies [30]—could further enhance fault sensitivity under extremely low signal-to-noise ratio conditions. This direction will be explored in future work.

Future work will focus on incorporating additional physical variables, such as flow and current signals, and extending the framework to online monitoring scenarios and more complex operating conditions. Future work will further investigate the influence of explicit noise injection on diagnostic performance, following recent studies on noise-enhanced intelligent fault diagnosis. For practical deployment, the proposed method requires only dual pressure sensors at the inlet and outlet ports of the valve, minimizing instrumentation overhead. Reliable diagnostic performance can be achieved with a limited number of fault samples, making the approach suitable for early-stage quality inspection. The framework can be readily integrated into existing SCADA or IIoT systems as a lightweight feature-extraction and decision module.

In conclusion, the proposed method presents a promising solution for small-sample hydraulic fault diagnosis, offering a balance of interpretability, computational efficiency, and industrial deployability. By leveraging physical simulation solely for feature design, rather than data generation, the framework avoids the need for large synthetic datasets and complex models. This design makes it particularly suitable for industrial applications where real-time applicability, transparency, and small-sample efficiency are essential.

## Figures and Tables

**Table 1:** Methodological comparison between representative small-sample fault diagnosis approaches and the proposed framework.

Method	Simulation Usage	Target System	Diagnostic Strategy	Feature Source
Digital twin-guided physical–virtual denoising [26]	Digital twin generates virtual signals for denoising	Rolling element bearing	Deep learning-based fault detection	Signal representation learned from virtual-real data
Digital twin cross-domain adaptation [27]	Digital twin generates simulated datasets for domain adaptation	Rolling element bearing	Cross-domain deep learning	Feature representation learned through domain adaptation
Data-driven intelligent diagnostic approaches [29,30,31]	Not required	Industrial equipment/actuators	Small-sample learning or feature-based classification	Data-driven feature representation
Proposed method	Simulation used for mechanism analysis	Hydraulic directional control valve	Classical machine learning classifiers	Physically interpretable pressure-difference features

**Table 2:** Simulation Parameters.

Maximum Flow Coefficient	0.7
Spring stiffness	10 N/mm
Damper mass	1 kg
Static friction	10 N
Coulomb friction	10 N
Static friction coefficient	100 N/(m/s)

**Table 3:** Technical specifications of the experimental setup.

Category	Key Parameters & Specifications
Test Rig	Max Pressure: 32 MPa; Max Flow: 150 L/min; Exp. Pressure: 21 MPa
DUT (Valves)	Type A: Rexroth 4WE10H3X (Ref); Type B: Hydronor 4WE10H3X (Domestic); Rated Flow: ~120 L/min
Sensors	Model: Danfoss MBS 3050; Range: 0–10 MPa; Accuracy: ±0.5% FS; Signal: 4–20 mA
Data Acq.	Sampling: 1 kHz; Cycle: 60 s; Samples: 90 cycles/cond.

**Table 4:** Representative experimental measurement data for one sample per fault condition. Each sample consists of the first 1000 points from synchronized pressure signals at the inlet (PS1) and outlet (PS2) ports.

Sample ID	Fault Type	Spring Stiffness (% of Nominal)	Orifice Blockage (%)	Coil Current (A)	Mean (P1) (MPa)	Mean (P2) (MPa)	Mean (ΔP) (MPa)	Std (ΔP) (MPa)
EXP-N	Normal	100	0	1.20	18.992	14.205	4.787	0.018
EXP-S	Spring Degradation	60	0	1.20	18.976	14.145	4.831	0.032
EXP-B	Orifice Blockage	100	50	1.20	19.005	11.548	7.457	0.021
EXP-C	Electromagnetic Degradation	100	0	0.72	18.963	14.188	4.775	0.028

**Table 5:** Training Results of Different Models Using Only DTW Features.

Model	Accuracy	Precision	Recall	F1 Score
RandomForest	0.9106	0.9105	0.9106	0.91
K-Nearest Neighbors	0.8269	0.8261	0.8269	0.8264
SVM	0.3894	0.3565	0.3894	0.3307
LogisticRegression	0.3708	0.3793	0.3708	0.3383

**Table 6:** Training Results of Different Models Using Manhattan Distance + DTW.

Model	Accuracy	Precision	Recall	F1 Score
RandomForest	0.8157	0.8158	0.8157	0.8153
K-Nearest Neighbors	0.7801	0.779	0.7801	0.7794
SVM	0.3769	0.3413	0.3769	0.3113
LogisticRegression	0.3745	0.4097	0.3745	0.2971

**Table 7:** Training results of different models using Manhattan distance + statistical analysis.

Model	Accuracy	Precision	Recall	F1 Score
RandomForest	0.9977	0.9977	0.9977	0.9977
K-Nearest Neighbors	0.8963	0.8967	0.8963	0.8963
SVM	0.3917	0.3593	0.3917	0.3372
LogisticRegression	0.6153	0.6343	0.6153	0.5739

**Table 8:** Training Results of Different Models Using Euclidean Distance + Statistical Features.

Model	Accuracy	Precision	Recall	F1 Score
RandomForest	0.9977	0.9977	0.9977	0.9977
KNeighbors	0.8963	0.8967	0.8963	0.8963
SVM	0.3917	0.3593	0.3917	0.3372
LogisticRegression	0.6153	0.6343	0.6153	0.5739

**Table 9:** Training Results of Different Models Using Euclidean Distance + DTW.

Model	Accuracy Rate	Precision	Recall	F1 Score
RandomForest	0.8157	0.8158	0.8157	0.8153
K-Nearest Neighbors	0.7801	0.779	0.7801	0.7794
SVM	0.3769	0.3413	0.3769	0.3113
LogisticRegression	0.3745	0.4097	0.3745	0.2971

**Table 10:** Overfitting Analysis Data for Valve Classification Using Manhattan Distance + Statistical Features and Euclidean Distance + Statistical Features.

Overfitting Analysis	Manhattan + Statistical	Euclidean Distance + Statistical
Number of Samples	10,800	10,800
Feature Dimension	5	5
Sample/Feature Ratio	2160.00	2160.00
Training set average accuracy	1.0000 ± 0.0000	1.0000 ± 0.0000
Validation set average accuracy	0.9979 ±0.0008	0.9973 ± 0.0014
Overfitting gap	0.0021	0.0027
Proportion of unstable features (CV > 0.3)	0.00	0.00
Top 5 most important features and their coefficients of variation	Feature 4: Importance = 0.4008, CV = 0.0111	Feature 4: Importance = 0.3663, CV = 0.0183
	Feature 1: Importance = 0.2780, CV = 0.0097	Feature 1: Importance = 0.2797, CV = 0.0141
	Feature 3: Importance = 0.1573, CV = 0.0313	Feature 0: Importance = 0.1944, CV = 0.0304
	Feature 0: Importance = 0.1440, CV = 0.0405	Feature 3: Importance = 0.1397, CV = 0.0200
	Feature 2: Importance = 0.0200, CV = 0.0909	Feature 2: Importance = 0.0199, CV = 0.0764
Number of PCA components retaining 90% variance	2	2
Average training set accuracy after PCA	1.0000	1.0000
Average accuracy on validation set after PCA	0.8477	0.8583
Overfitting gap after PCA	0.1523	0.1416

**Table 11:** Comparison between the proposed method and simulation-enhanced diagnosis frameworks.

Aspect	Proposed Method	Ning et al. [27]
Simulation Role	Offline mechanism analysis only; guides feature design	Online synthetic data generator for training.
Data Used for Training	Real measured signals only (limited samples).	Synthetic data (source) + real data (target, for adaptation).
Model Type	Classical ML (e.g., SVM, RF); interpretable.	Deep adversarial network (21+ layers); black-box.
Small-Sample Strategy	Physics-informed feature engineering.	Data augmentation via high-fidelity simulation.
Computational Cost	Low; suitable for edge deployment.	High; >3× longer training time reported.
Dependence on Simulation Fidelity	Weak; robust to modeling inaccuracies.	Strong; requires domain adaptation to close “reality gap”.

## Data Availability

The data presented in this study were obtained from a proprietary hydraulic test bench and are not publicly available due to confidentiality agreements with the collaborating enterprise. Reasonable requests for access may be directed to Changzheng TianMin Company.

## Supplementary Materials

The following supporting information can be downloaded at: https://www.mdpi.com/article/10.3390/s26072052/s1.
