# Supplementary figures and images for: Simulation-Guided Interpretable Fault Diagnosis of Hydraulic Directional Control Valves Under Limited Fault Data Conditions
Source: Sensors (Basel). 2026 Mar 25;26(7):2052. doi: 10.3390/s26072052 (PMC13074784; doi:10.3390/s26072052)

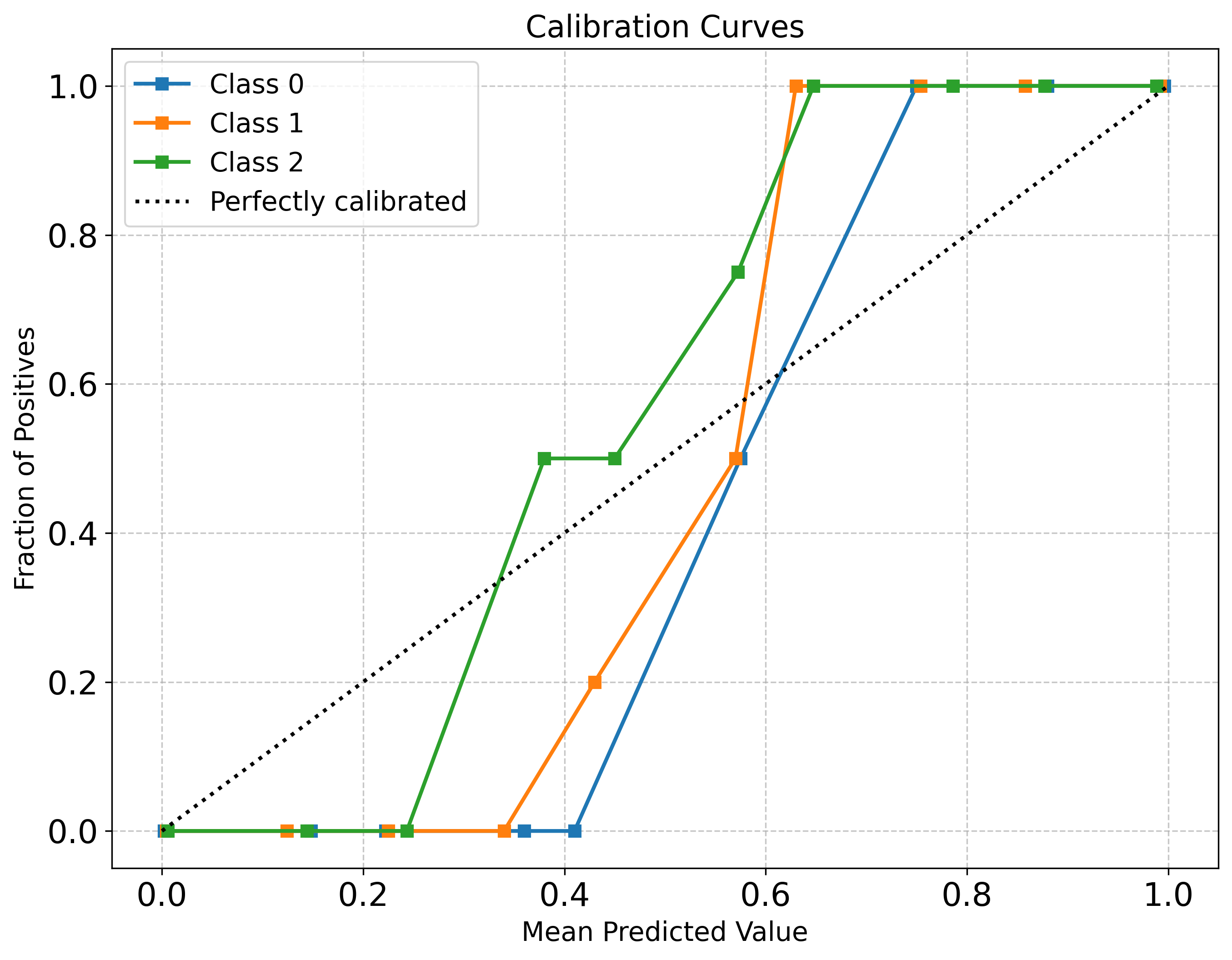

Supplement: Supplementary file 1 [file sensors-26-02052-s001.zip › Enhanced_Overfitting_Analysis_Euclidean+Stats_Valve/Calibration_Curves.png]

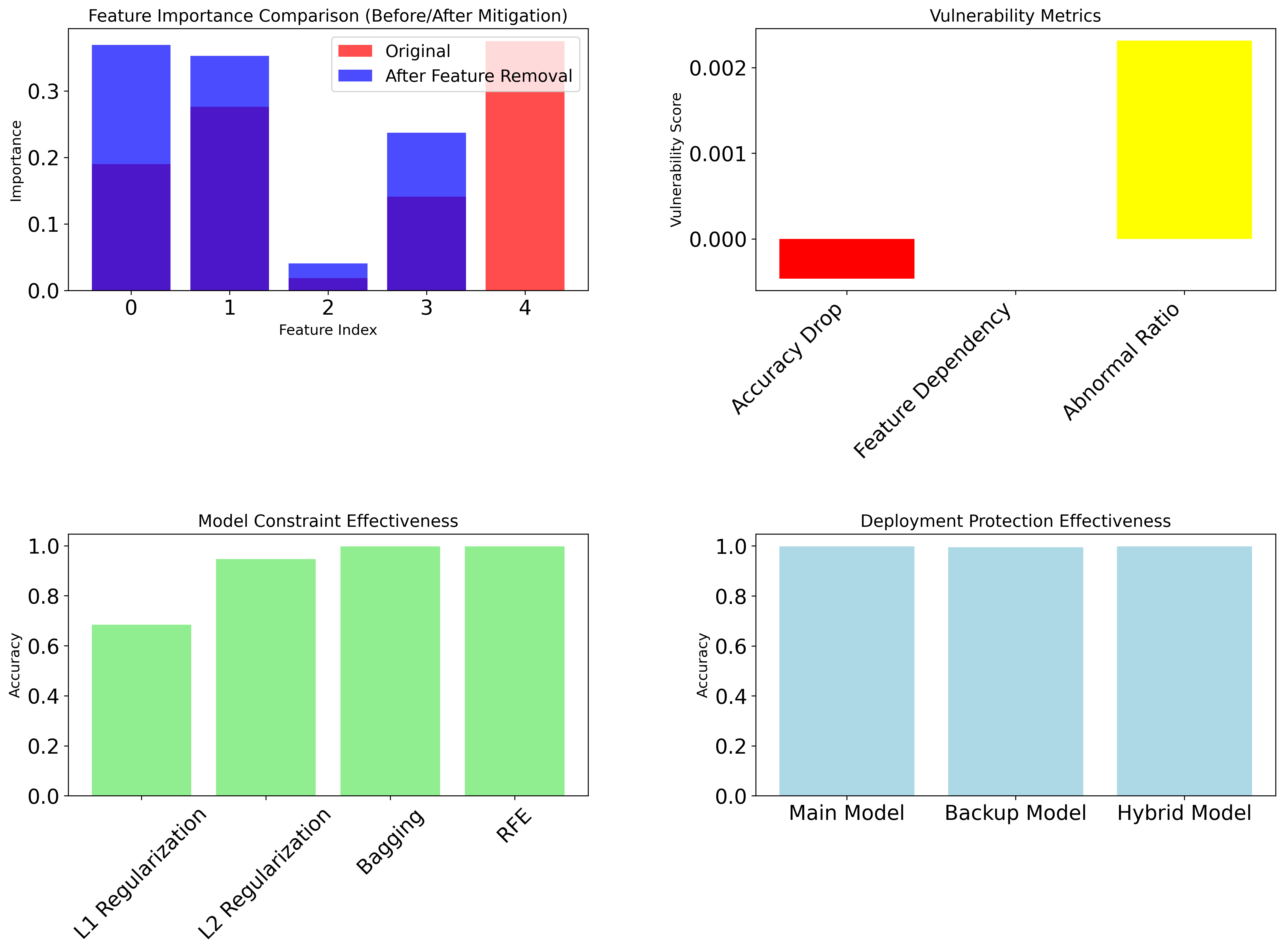

Supplement: Supplementary file 1 [file sensors-26-02052-s001.zip › Enhanced_Overfitting_Analysis_Euclidean+Stats_Valve/Comprehensive_Vulnerability_Analysis.png]

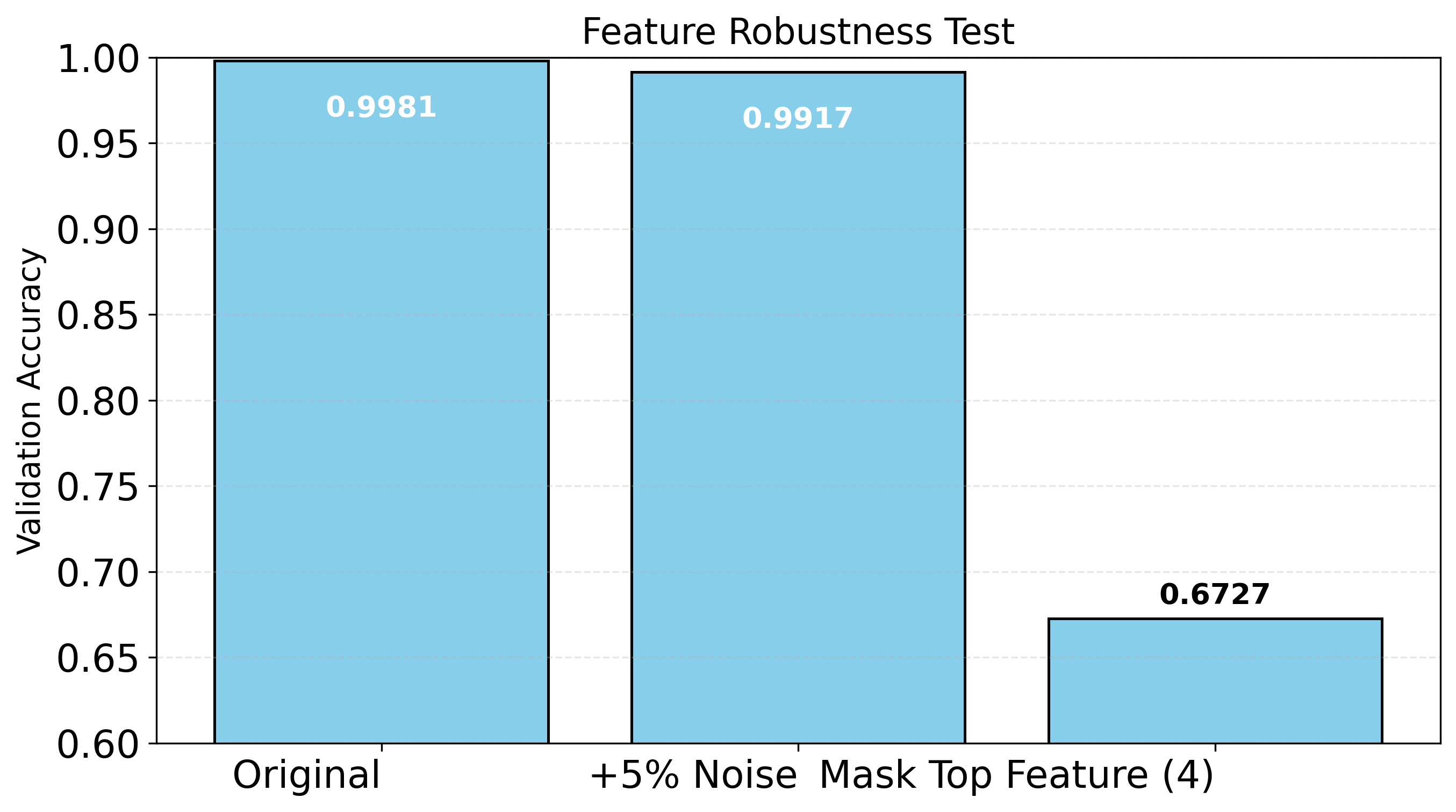

Supplement: Supplementary file 1 [file sensors-26-02052-s001.zip › Enhanced_Overfitting_Analysis_Euclidean+Stats_Valve/Feature_Robustness.png]

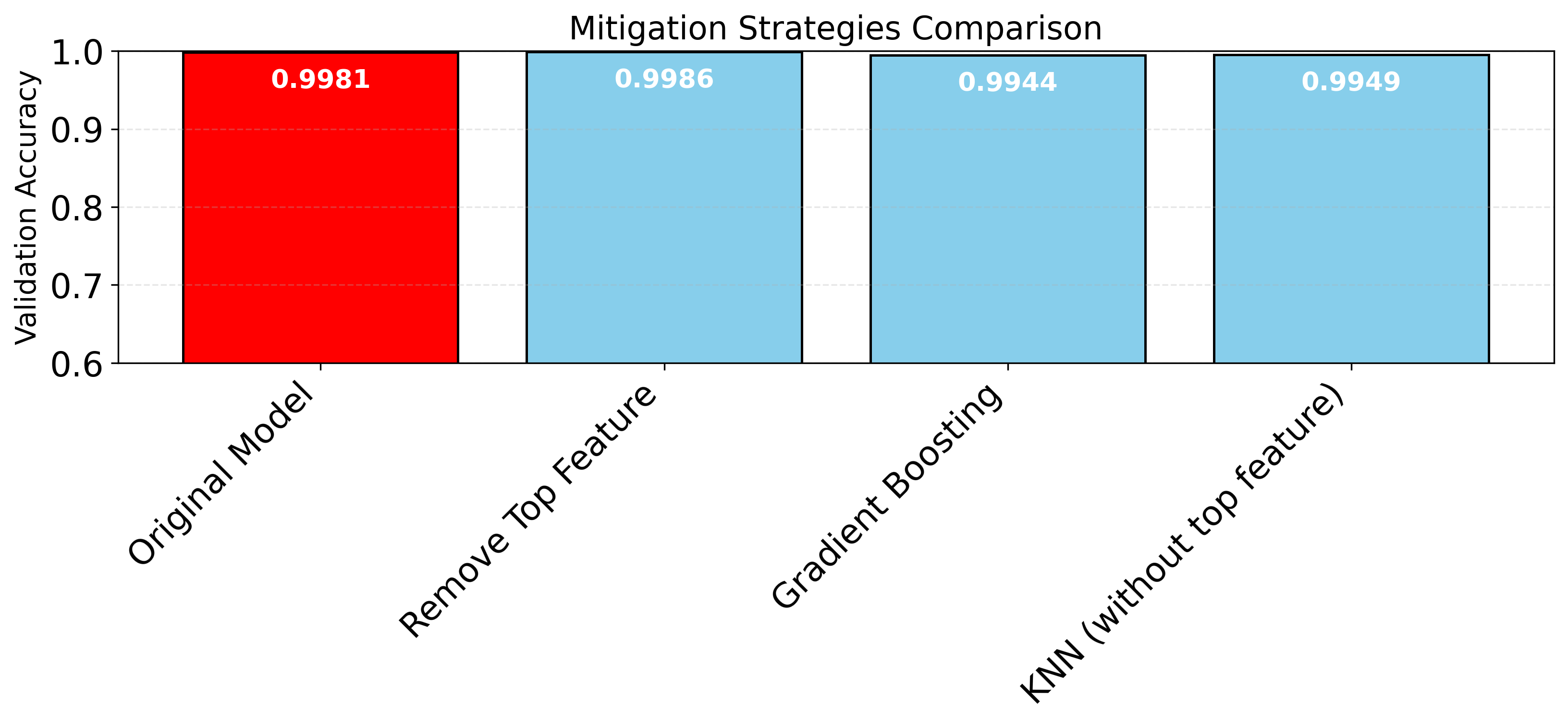

Supplement: Supplementary file 1 [file sensors-26-02052-s001.zip › Enhanced_Overfitting_Analysis_Euclidean+Stats_Valve/Mitigation_Strategies.png]

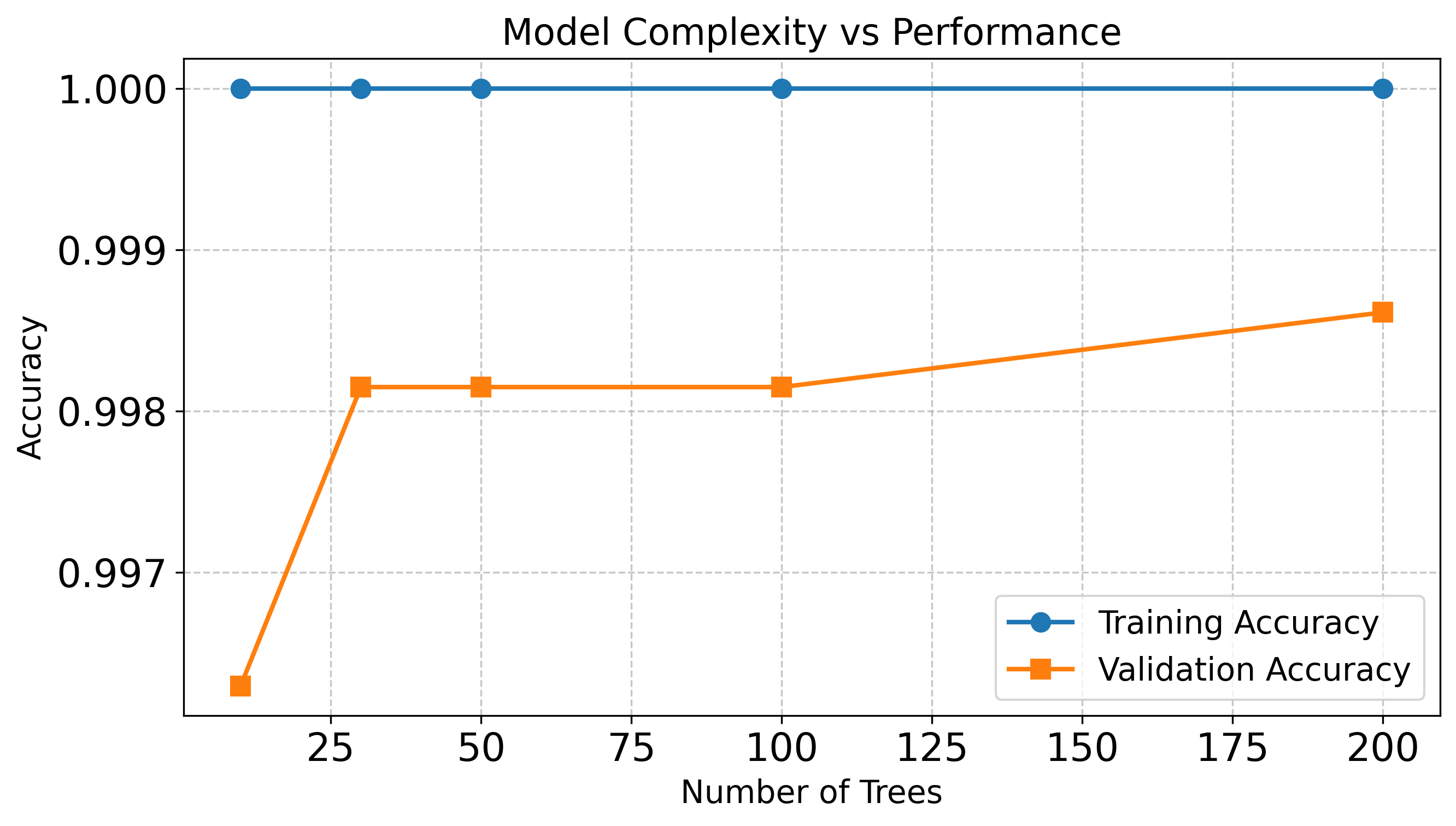

Supplement: Supplementary file 1 [file sensors-26-02052-s001.zip › Enhanced_Overfitting_Analysis_Euclidean+Stats_Valve/Model_Complexity_Ablation.png]

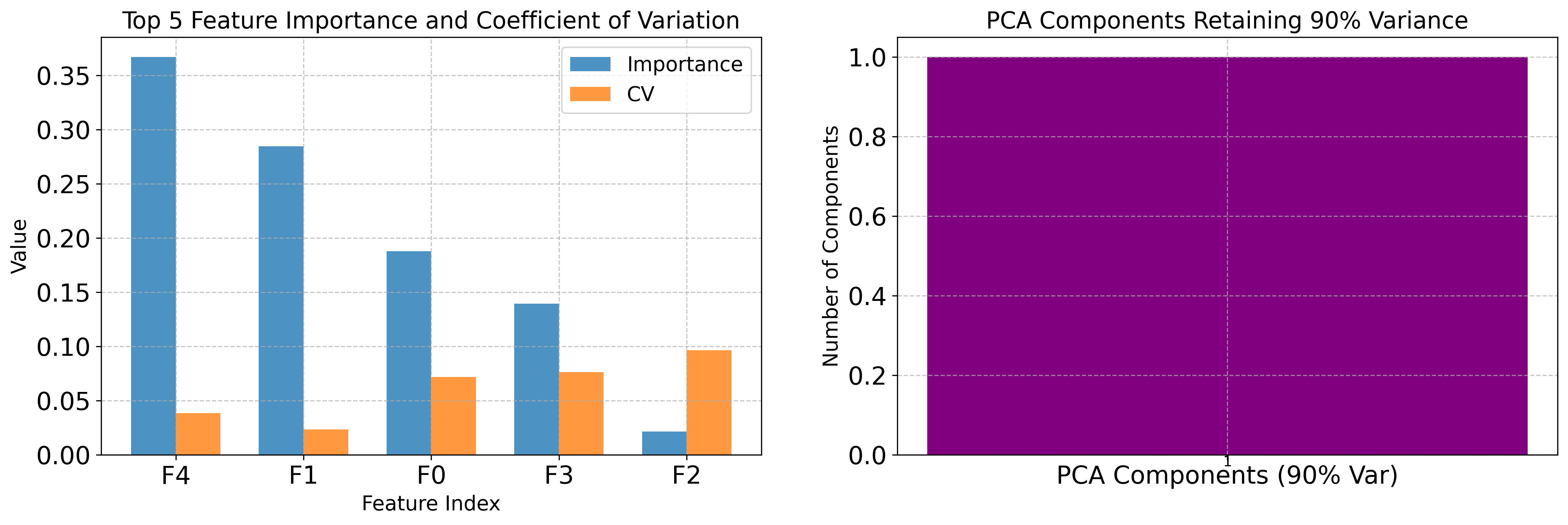

Supplement: Supplementary file 1 [file sensors-26-02052-s001.zip › Enhanced_Overfitting_Analysis_Euclidean+Stats_Valve/PCA_CV_Analysis_Features.png]

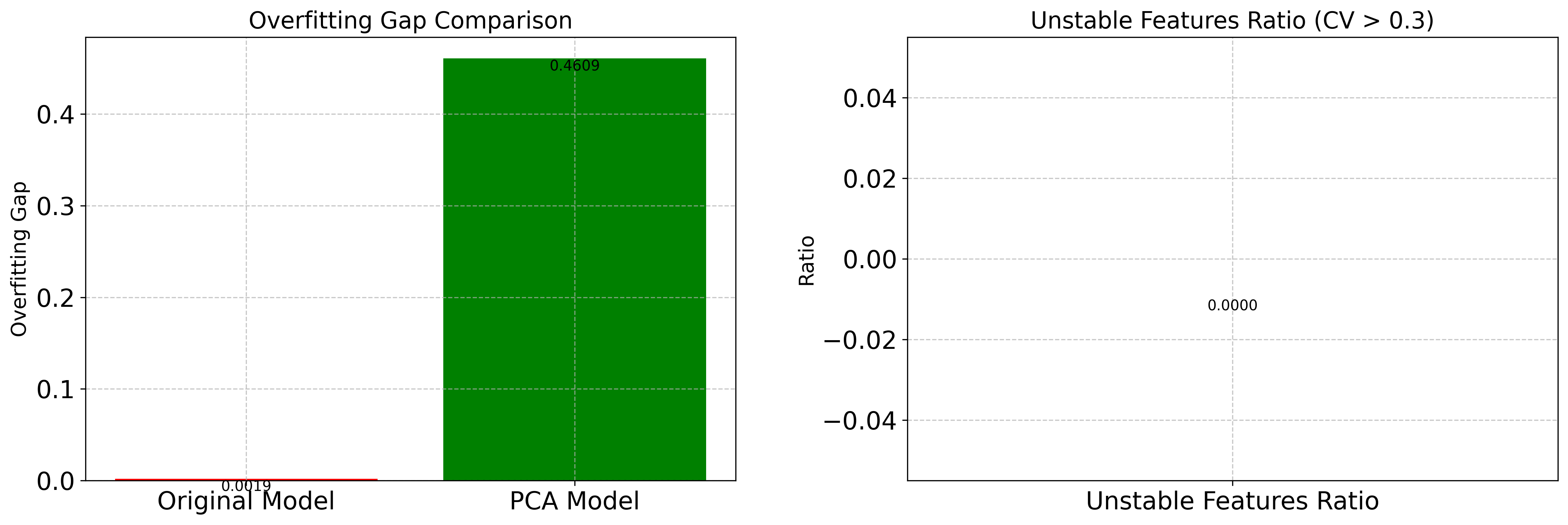

Supplement: Supplementary file 1 [file sensors-26-02052-s001.zip › Enhanced_Overfitting_Analysis_Euclidean+Stats_Valve/PCA_CV_Analysis_Overfitting.png]

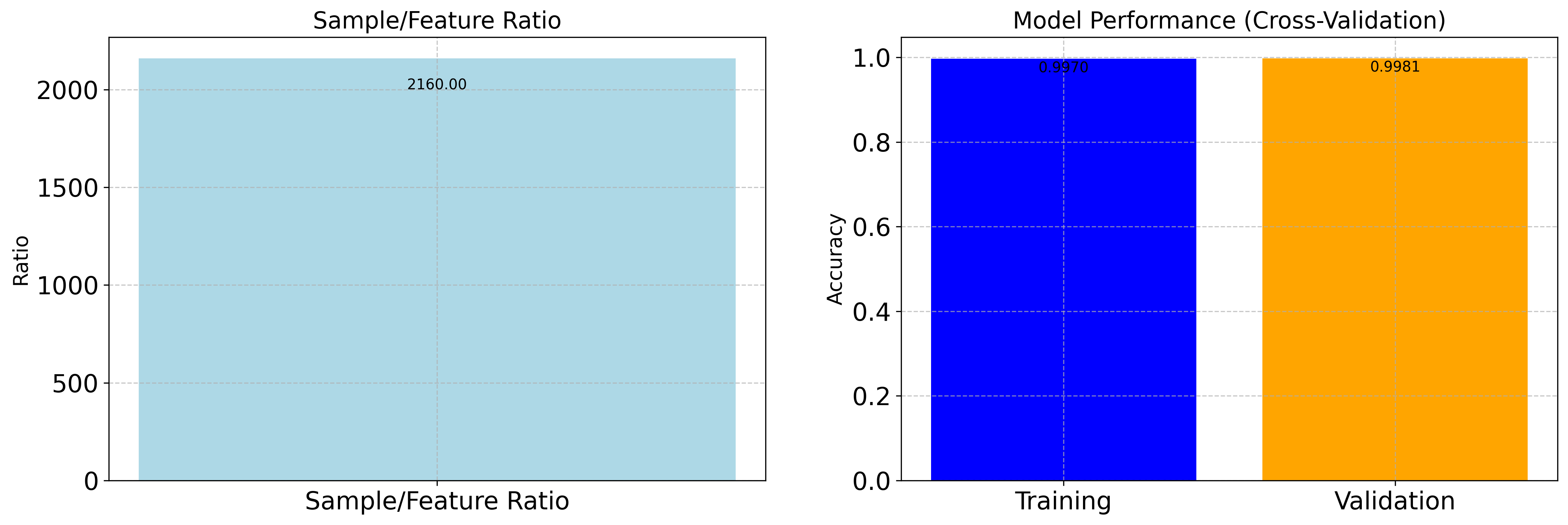

Supplement: Supplementary file 1 [file sensors-26-02052-s001.zip › Enhanced_Overfitting_Analysis_Euclidean+Stats_Valve/PCA_CV_Analysis_Performance.png]

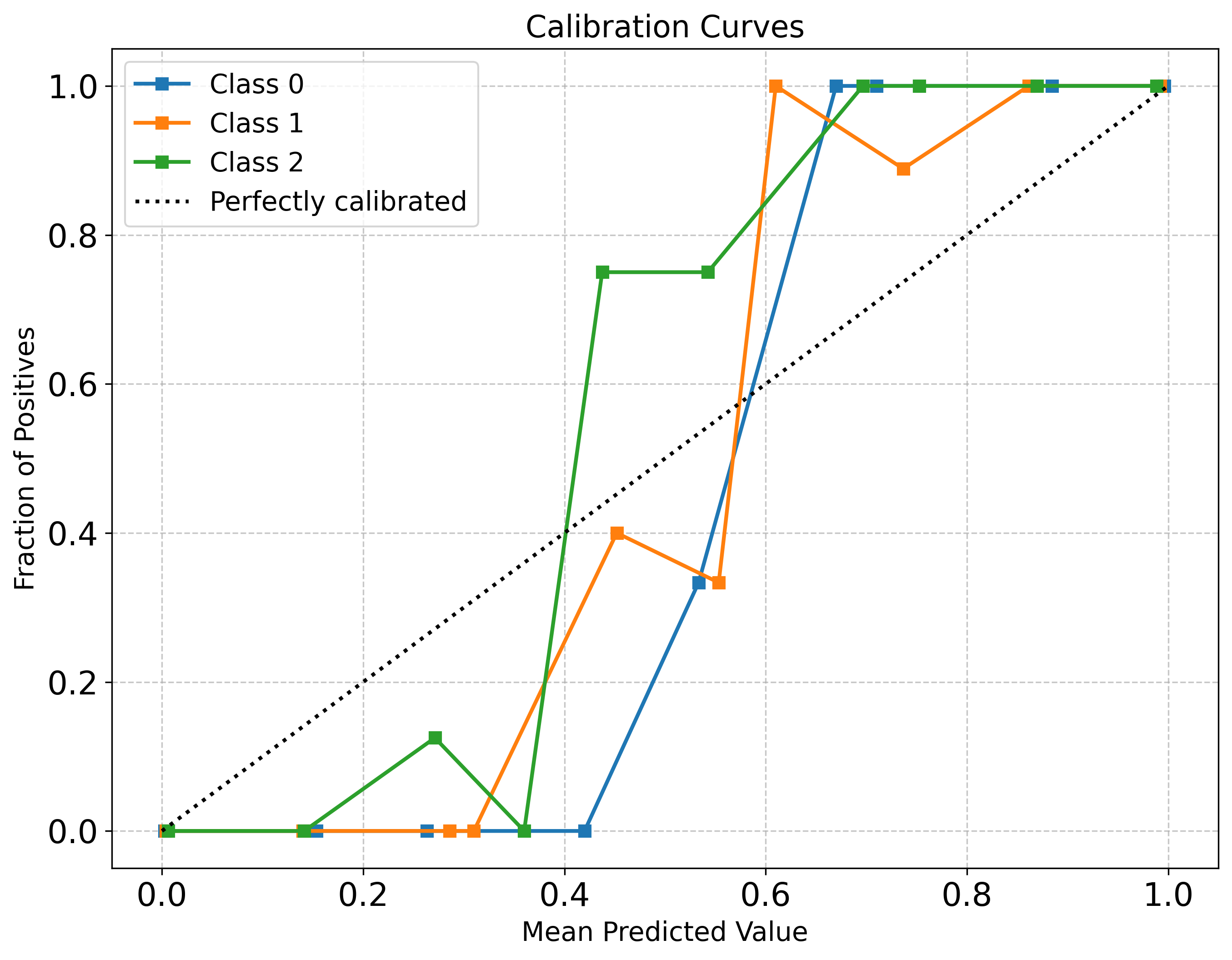

Supplement: Supplementary file 1 [file sensors-26-02052-s001.zip › Enhanced_Overfitting_Analysis_Manhattan+Stats_Valve/Calibration_Curves.png]

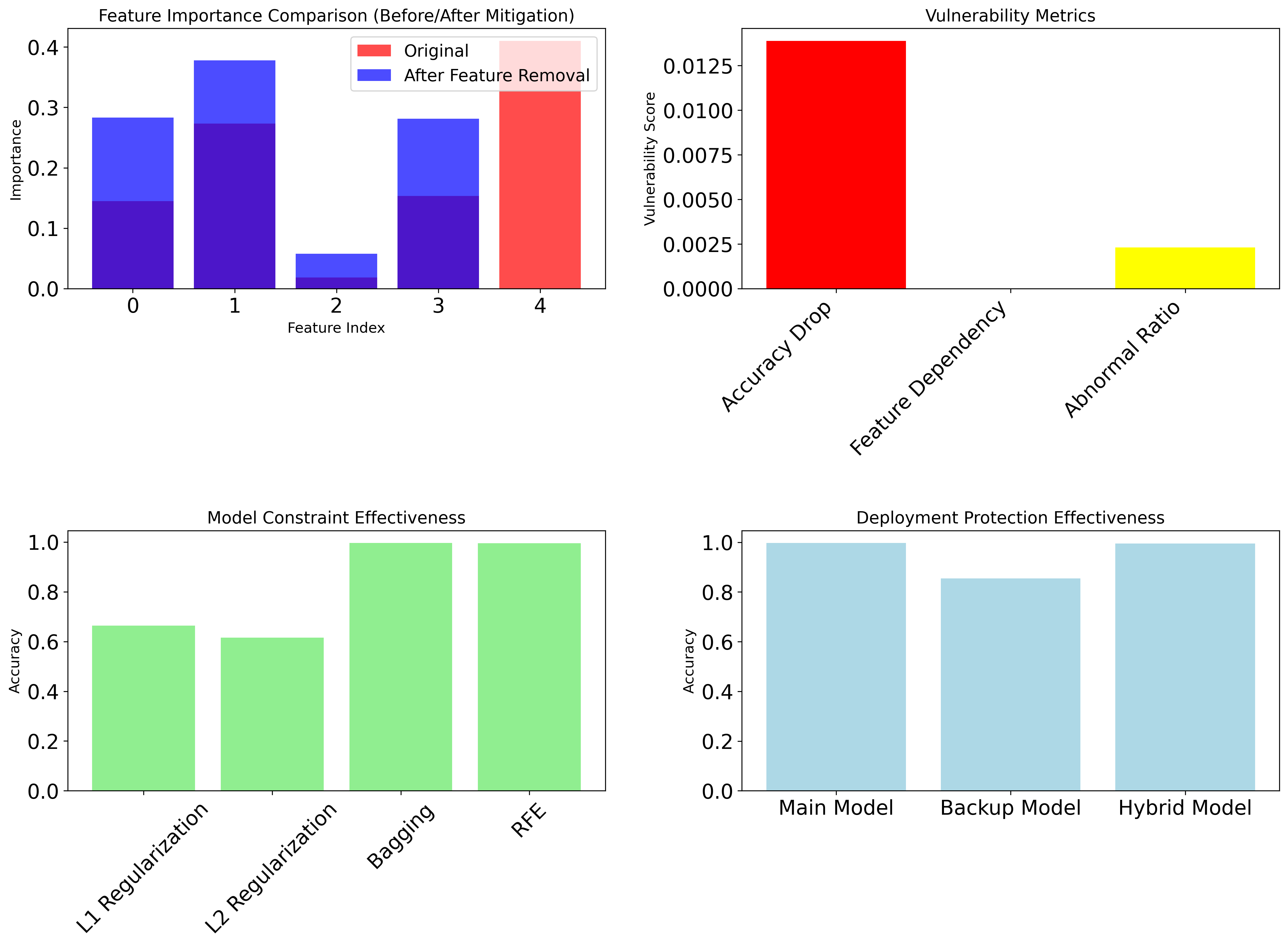

Supplement: Supplementary file 1 [file sensors-26-02052-s001.zip › Enhanced_Overfitting_Analysis_Manhattan+Stats_Valve/Comprehensive_Vulnerability_Analysis.png]

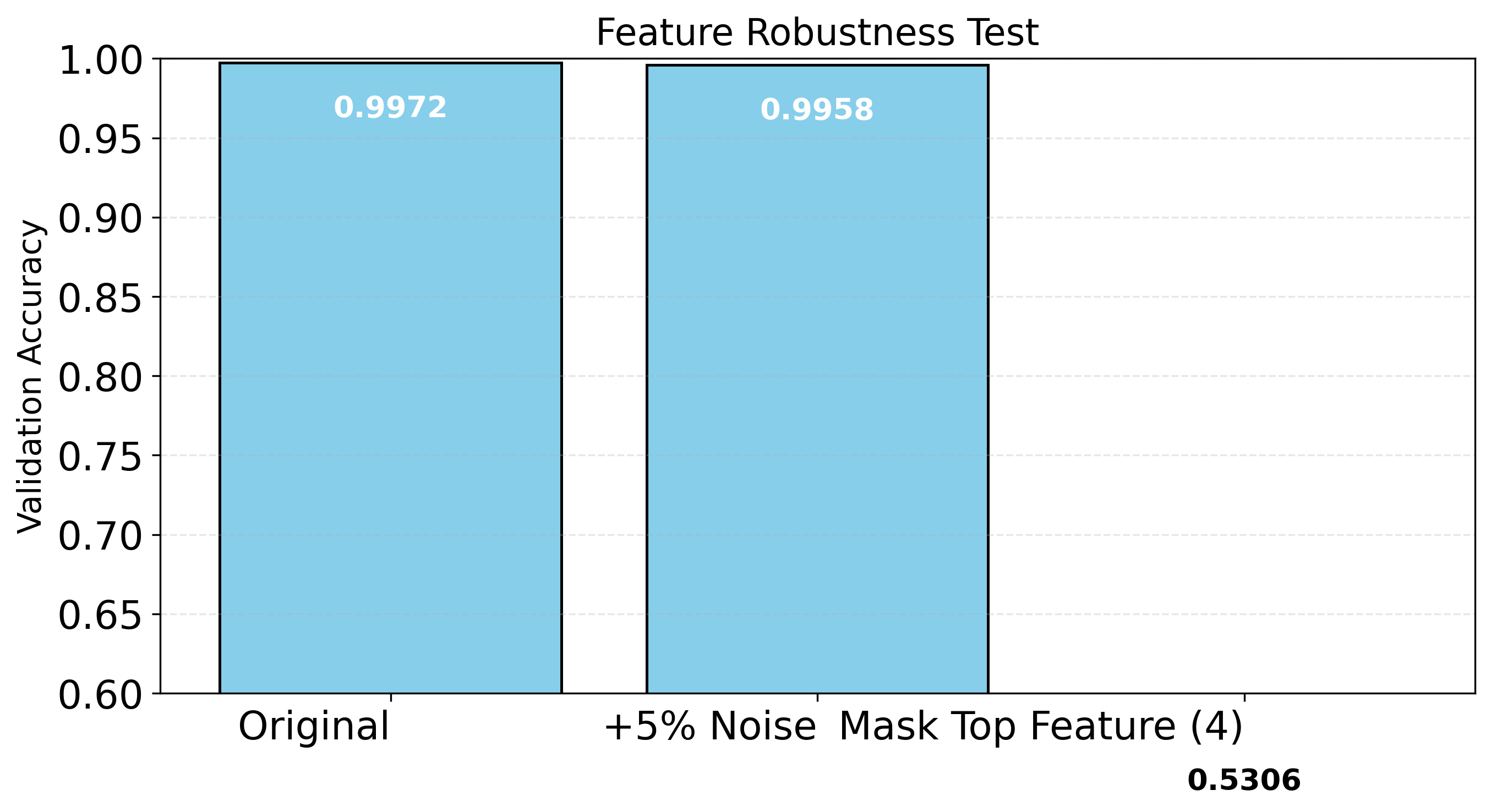

Supplement: Supplementary file 1 [file sensors-26-02052-s001.zip › Enhanced_Overfitting_Analysis_Manhattan+Stats_Valve/Feature_Robustness.png]

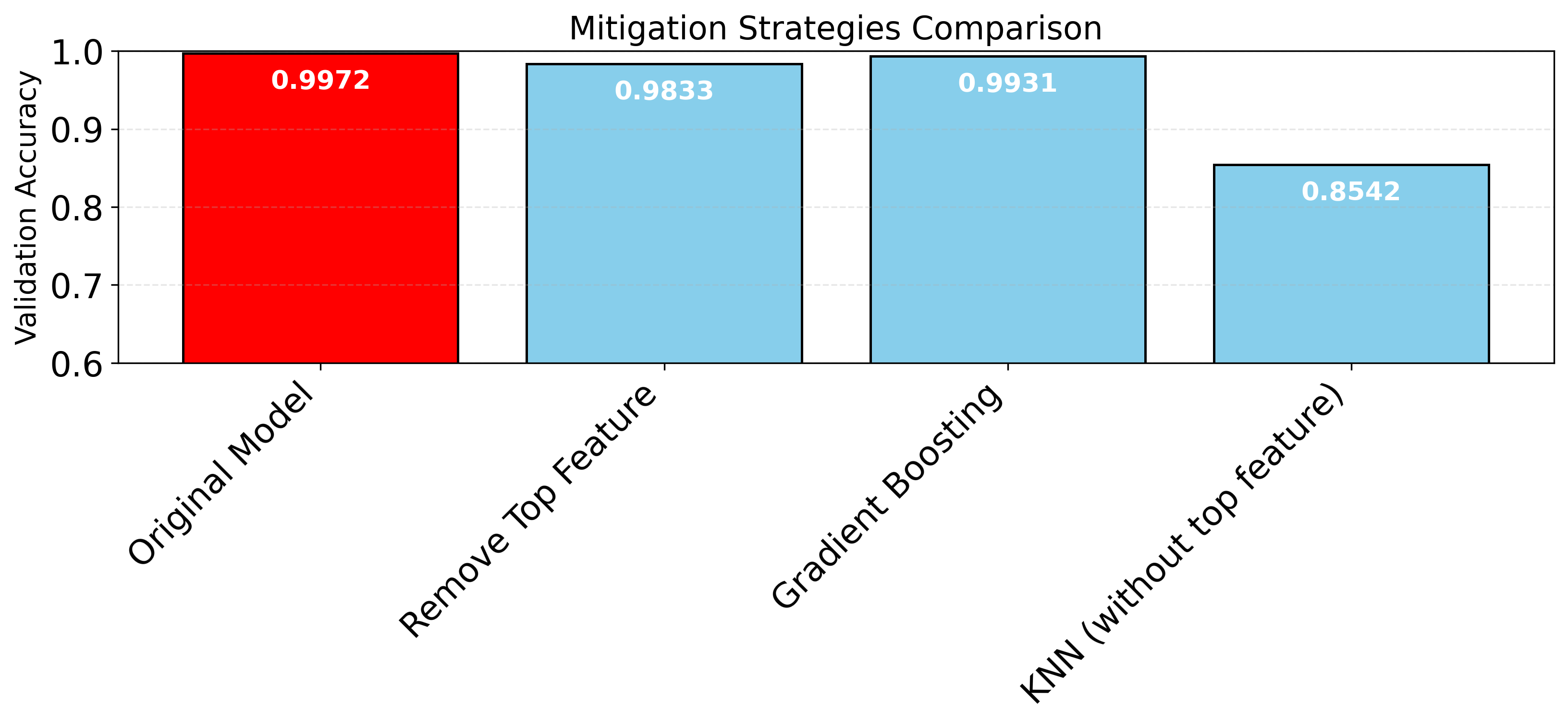

Supplement: Supplementary file 1 [file sensors-26-02052-s001.zip › Enhanced_Overfitting_Analysis_Manhattan+Stats_Valve/Mitigation_Strategies.png]

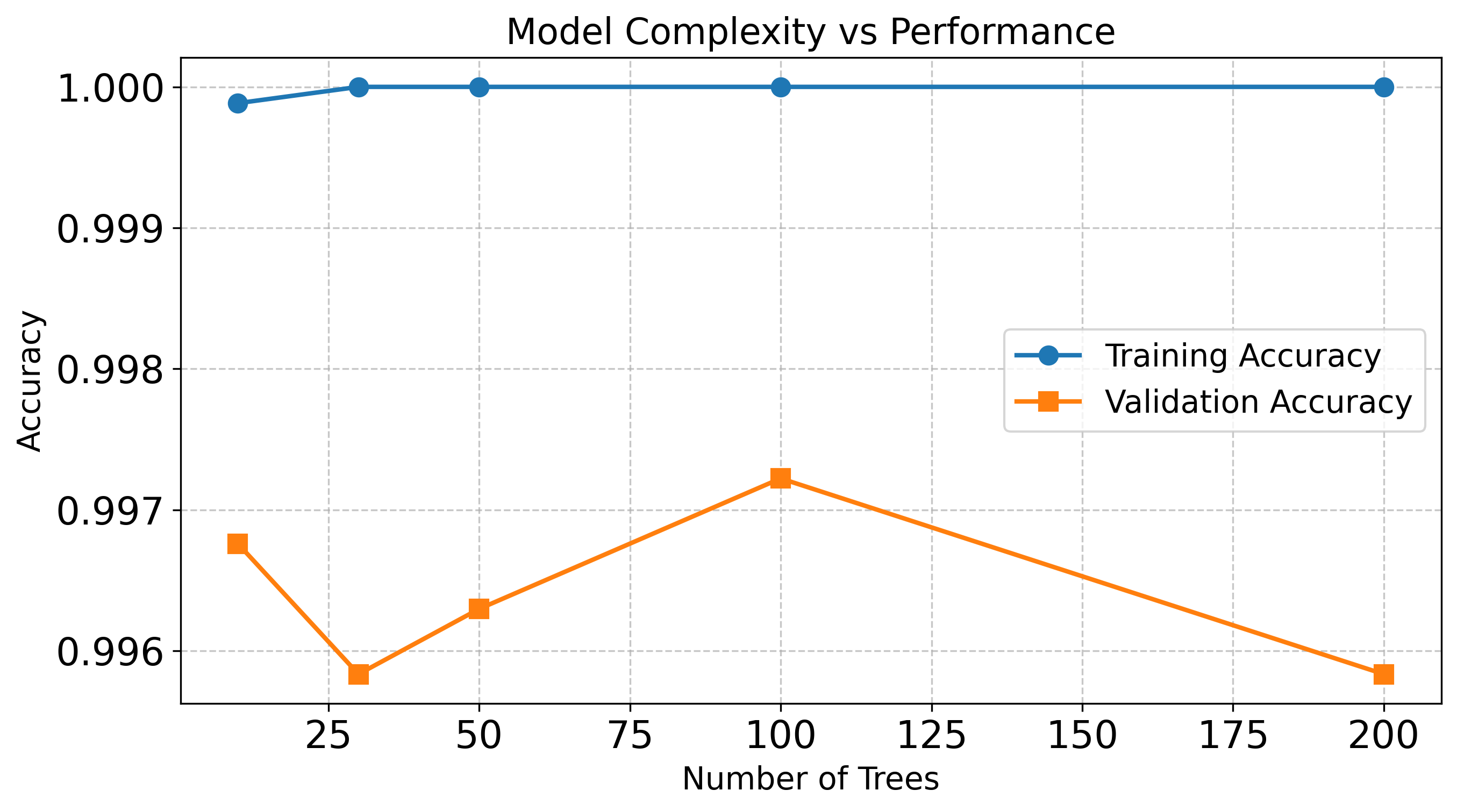

Supplement: Supplementary file 1 [file sensors-26-02052-s001.zip › Enhanced_Overfitting_Analysis_Manhattan+Stats_Valve/Model_Complexity_Ablation.png]

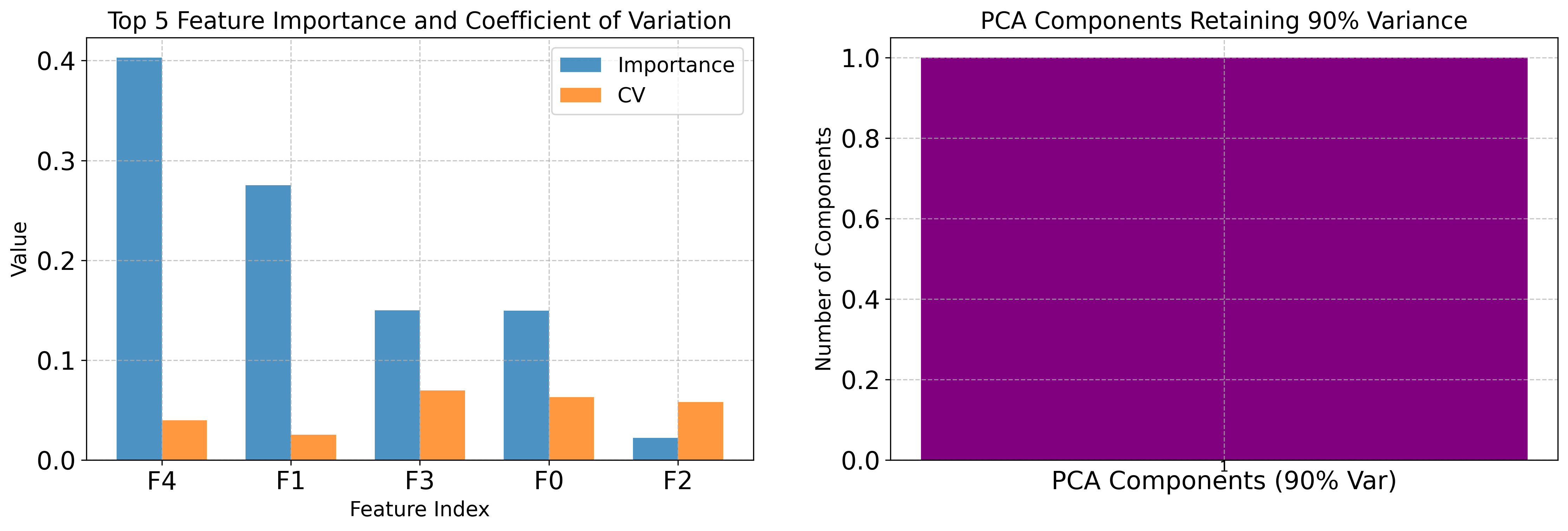

Supplement: Supplementary file 1 [file sensors-26-02052-s001.zip › Enhanced_Overfitting_Analysis_Manhattan+Stats_Valve/PCA_CV_Analysis_Features.png]

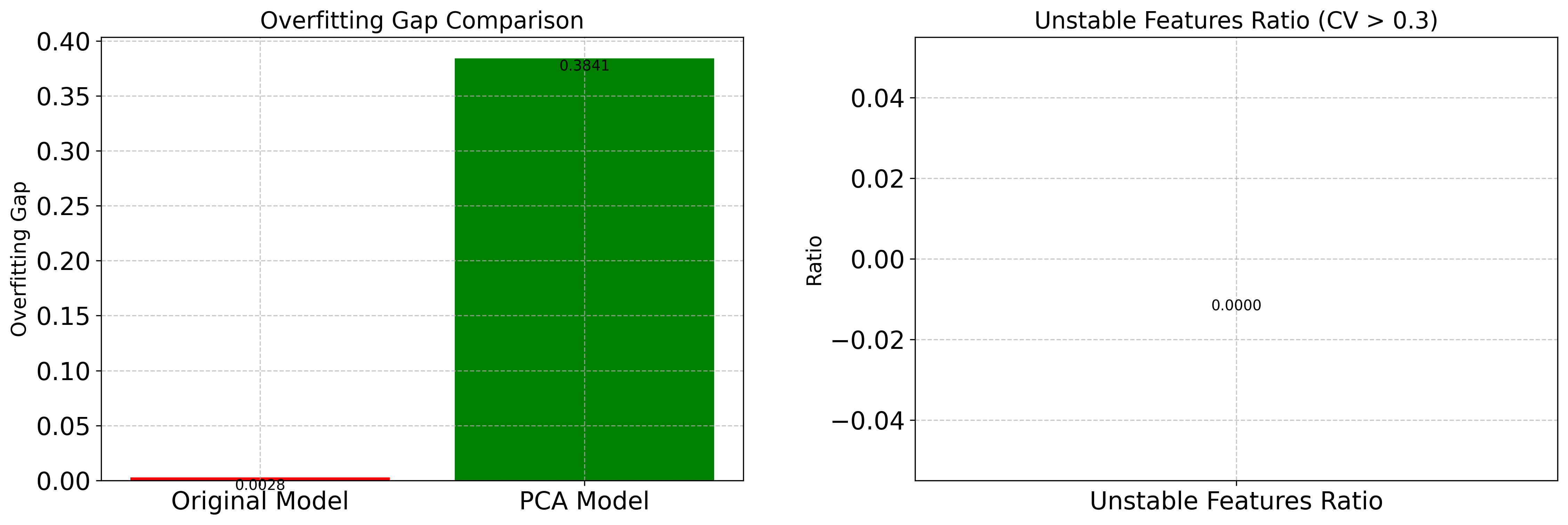

Supplement: Supplementary file 1 [file sensors-26-02052-s001.zip › Enhanced_Overfitting_Analysis_Manhattan+Stats_Valve/PCA_CV_Analysis_Overfitting.png]

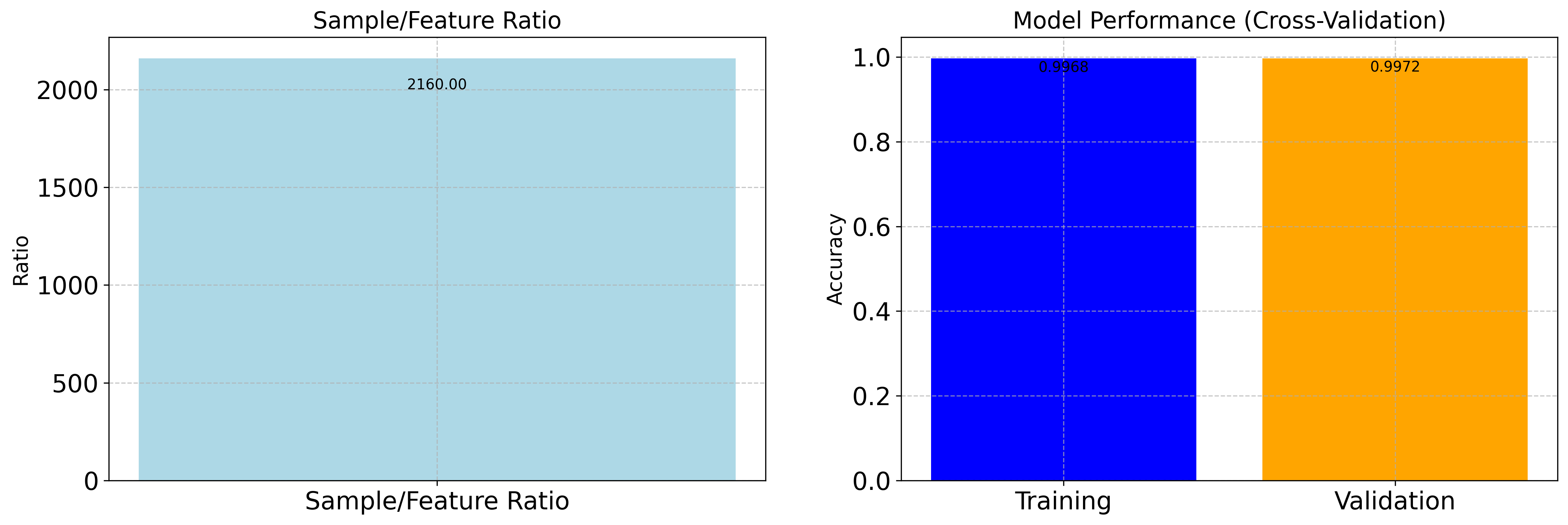

Supplement: Supplementary file 1 [file sensors-26-02052-s001.zip › Enhanced_Overfitting_Analysis_Manhattan+Stats_Valve/PCA_CV_Analysis_Performance.png]
